# A simple and robust nanosystem for photoacoustic imaging of bladder cancer based on α5β1-targeted gold nanorods

**DOI:** 10.1186/s12951-023-02028-5

**Published:** 2023-08-27

**Authors:** Massimo Alfano, Elisa Alchera, Angelina Sacchi, Alessandro Gori, Giacomo Quilici, Irene Locatelli, Chiara Venegoni, Roberta Lucianò, Anna Maria Gasparri, Barbara Colombo, Giulia Taiè, Jithin Jose, Paolo Armanetti, Luca Menichetti, Giovanna Musco, Andrea Salonia, Angelo Corti, Flavio Curnis

**Affiliations:** 1 Unit of Urology, URI, Division of Experimental Oncology, IRCCS San Raffaele Scientific Institute, Milan, Italy; 2 Tumor Biology and Vascular Targeting Unit, Division of Experimental Oncology, IRCCS San Raffaele Scientific Institute, Via Olgettina 58, 20132 Milan, Italy; 3 Istituto di Scienze e Tecnologie Chimiche, C.N.R., Milan, Italy; 4 Biomolecular NMR Laboratory, IRCCS San Raffaele Scientific Institute, Milan, Italy; 5 Department of Pathology, IRCCS San Raffaele Scientific Institute, Milan, Italy; 6 FUJIFILM Visualsonics Inc, Amsterdam, The Netherlands; 7 Institute of Clinical Physiology, Italian National Research Council (CNR), Pisa, Italy; 8 Università Vita-Salute San Raffaele, Milan, Italy

**Keywords:** *Iso*DGR motif, α5β1 integrin, Gold nanorods, Photoacoustic imaging, Bladder cancer

## Abstract

**Background:**

Early detection and removal of bladder cancer in patients is crucial to prevent tumor recurrence and progression. Because current imaging techniques may fail to detect small lesions of in situ carcinomas, patients with bladder cancer often relapse after initial diagnosis, thereby requiring frequent follow-up and treatments.

**Results:**

In an attempt to obtain a sensitive and high-resolution imaging modality for bladder cancer, we have developed a photoacoustic imaging approach based on the use of PEGylated gold nanorods (GNRs) as a contrast agent, functionalized with the peptide cyclic [CphgisoDGRG] (Iso4), a selective ligand of α5β1 integrin expressed by bladder cancer cells. This product (called GNRs@PEG-Iso4) was produced by a simple two-step procedure based on GNRs activation with lipoic acid-polyethyleneglycol(PEG-5KDa)-maleimide and functionalization with peptide Iso4. Biochemical and biological studies showed that GNRs@PEG-Iso4 can efficiently recognize purified integrin α5β1 and α5β1-positive bladder cancer cells. GNRs@PEG-Iso4 was stable and did not aggregate in urine or in 5% sodium chloride, or after freeze/thaw cycles or prolonged exposure to 55 °C, and, even more importantly, do not settle after instillation into the bladder. Intravesical instillation of GNRs@PEG-Iso4 into mice bearing orthotopic MB49-Luc bladder tumors, followed by photoacoustic imaging, efficiently detected small cancer lesions. The binding to tumor lesions was competed by a neutralizing anti-α5β1 integrin antibody; furthermore, no binding was observed to healthy bladders (α5β1-negative), pointing to a specific targeting mechanism.

**Conclusion:**

GNRs@PEG-Iso4 represents a simple and robust contrast agent for photoacoustic imaging and diagnosis of small bladder cancer lesions.

**Graphical Abstract:**

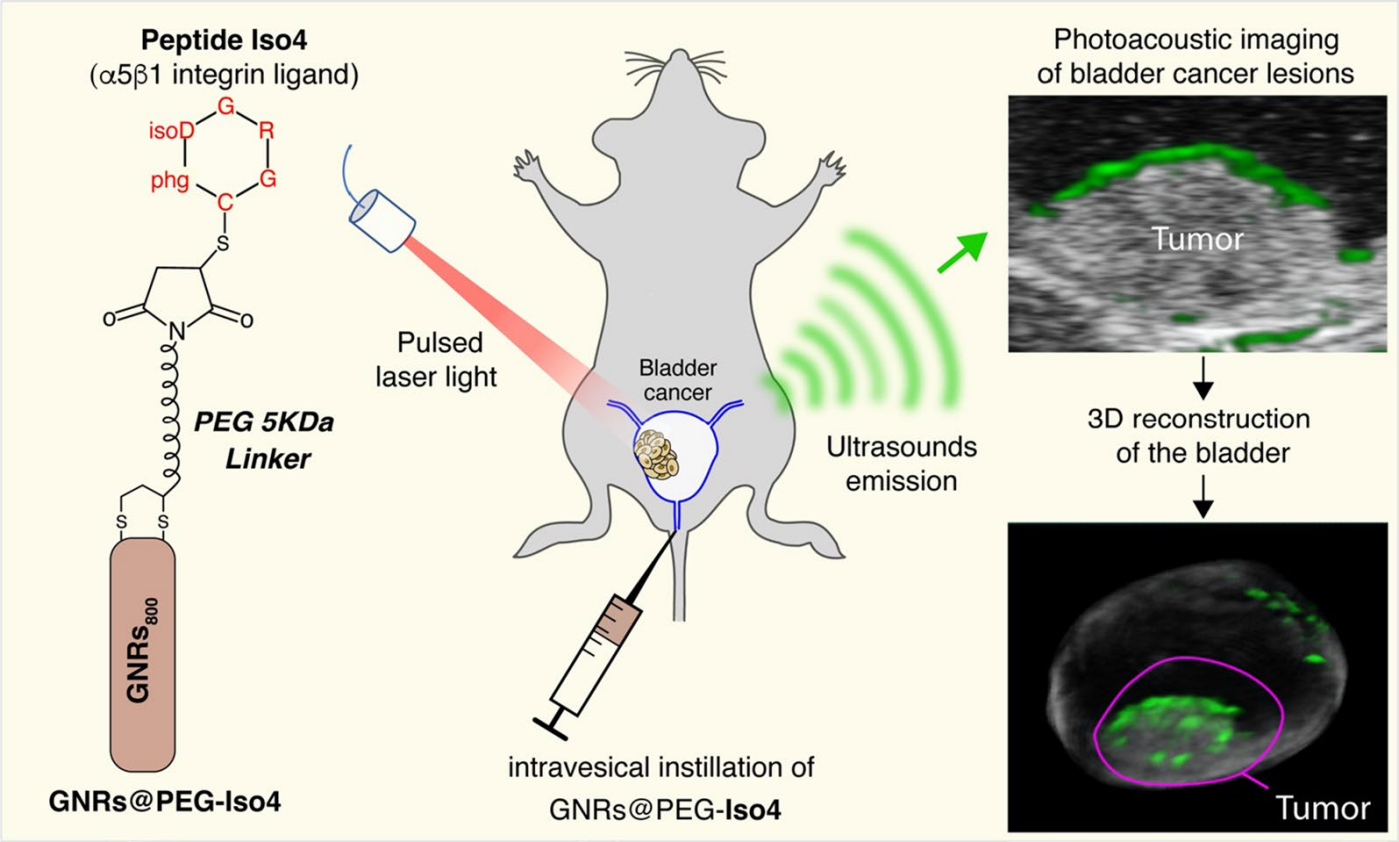

**Supplementary Information:**

The online version contains supplementary material available at 10.1186/s12951-023-02028-5.

## Background 

Urinary bladder cancer is one of the most common cancers worldwide, with > 0.5 million new cases and 0.2 million deaths per year [1]. At the time of diagnosis, approximately 75% of patients with bladder cancer have non-invasive bladder cancer (NMIBC), such as small flat lesions in the innermost layer of the urothelium (carcinoma *in situ*, *CIS*) or intraepithelial tumors confined to the mucosa (stage *Ta*) or tumors invading the lamina propria (stage *T1*) [2]. These tumors are surgically removed by TURBT (transurethral resection of bladder tumor) during cystoscopy with a resectoscope, followed by adjuvant therapy [3]. However, the bladder CIS, which usually consists of a small number of high-grade neoplastic cells, appears as a flat reddish area that may be missed or misinterpreted as an inflammatory lesion during cystoscopy examination. Poor detection of small lesions may occur also with other diagnostic techniques, such as those based on intravesical instillation of 5-aminolevulinic acid (followed by photodynamic imaging), ultrasound echography, computed tomography urography, multiparametric magnetic resonance imaging, or intravenous urography: indeed, all these techniques show limited diagnostic utility for CIS lesions 1–5 mm in size [4]. Thus, detection and management of patients with bladder CIS still represents a challenge in the onco-urological field [5, 6]. As a result, 40% of NMIBC patients have residual lesions after the initial TURBT [7], and, because of this diagnostic limitation, many patients with NMIBC frequently relapse after the initial treatment [3]. The limited therapeutic response in these patients leads to frequent and endless follow-up examinations and repeated treatments, worsening their quality of life [3]. Reducing the relapse rate in NMIBC is, therefore, a priority to improve the quality of life of these patients and the associated social costs [8, 9].

A unique feature of superficial bladder cancer is the possibility of delivering medical therapies, imaging, and theranostic compounds directly into the bladder *via* a catheter [3]. This approach can reduce systemic exposure to the treatments, thereby minimizing the toxic side effects of the administered compounds. According to this view, various studies have shown that gold nanoparticles, functionalized with different molecules and intravesically administered, can be used as contrast agents for photoacoustic imaging (PAI) or for photodynamic/photothermal therapy of bladder cancer lesions [10–17]. For example, we have recently demonstrated that bladder cancer lesions can be detected by intravesical instillation of gold nanorods (GNRs) decorated with chitosan and functionalized with a cyclic [CphgisoDGRG] peptide (Iso4), a ligand of α5β1 integrin [18], followed by imaging of bladder cancer lesion by a photoacoustic approach [15]. Remarkably, this nanoformulation (called GNRs@Chit-Iso4) allows the detection of extremely small bladder cancer lesions <0.5 mm by PAI in a murine model, i.e. with a level of detectability that cannot be achieved with conventional diagnostic approaches.

This imaging procedure requires that after intravesical injection to tumor-bearing mice, the GNRs@Chit-Iso4 nanoparticles are kept in suspension through the application of low-frequency ultrasound with a piezoelectric matrix array transducer [15], a procedure that may be difficult to translate in patients.

To overcome this problem, in the present work we tried to develop new α5β1-integrin targeted GNRs that do not settle upon intravesical instillation. In particular, we investigated the feasibility of a very simple procedure for the functionalization of PEGylated GNRs with Iso4, based on the use of lipoic acid-polyethyleneglycol (PEG, 5KDa)-maleimide as a crosslinking reagent, and analyzed the biochemical and biological properties of the resulting product.

We show that the product (called GNRs@PEG-Iso4) is robust and functional in terms of α5β1 integrin binding and tumor cell recognition, is stable in urine, does not aggregate after freezing or exposure to high temperatures, and does not settle after instillation in mice bladder. Furthermore, using the MB49-Luc orthotopic bladder cancer model, we show that these nanoparticles can be exploited for photoacoustic imaging of small bladder cancer lesions without the need of procedures that prevent nanoparticle settling in the bladder.

## Materials and methods

### Reagents and cell lines

Human serum albumin (HSA) (20% w/v Flexbumin, Baxalta, catalog #07-19-76-995); recombinant human α5β1 integrin (R&D System, catalog #3230-A5-050); Dulbecco's phosphate buffered saline (DPBS) with calcium and magnesium (DPBS/Ca/Mg) (Thermofischer, catalog #14080); gold nanorods NanoXact™ (NanoComposix, #GRCN800) in citrate buffer (GNRs); lipoic acid-polyethyleneglycol (PEG, 5KDa)-maleimide (CD Bioparticles, catalog #CDN1712); anti-polyethyleneglycol (PEG) rat monoclonal antibody (mAb) clone 26A04 (Abcam, catalog #ab94764); anti-β1 integrin antibody (clone HMβ1-1, Biolegend, catalog #1022019); anti-α5 integrin antibody (clone HMα5-1, Biolegend, catalog #103902); anti-β3 integrin monoclonal antibody (clone HMβ3-1, Biolegend, catalog #104302); anti-β5 integrin monoclonal antibody (clone KN52, eBioscience™, catalog #14-0497-82); anti-αv integrin monoclonal antibody (clone RMV-7, eBioscience™, catalog #14-0512-82), anti-αvβ6 monoclonal antibody (clone 10D5, Millipore, catalog #MAB2077Z); armenian hamster IgG Isotype Control (eBioscience™; catalog #14-4888-81); mouse IgG Isotype Control (clone MOPC-21, Sigma, catalog #M5284); rat IgG1 Isotype Control (clone eBRG1, eBioscience™, catalog #14-4301-81); Alexa Fluor™ 488-labeled goat anti-mouse secondary antibody (ThermoFisher, Catalog #A-11001); Alexa Fluor™ 488-labeled goat anti-hamster secondary antibody (ThermoFisher); Alexa Fluor 488-labeled goat anti-rat secondary antibody (ThermoFisher, catalog #A-11006); horseradish peroxidase (HRP)-labeled goat anti-rat-IgM antiserum (Sigma, catalog #SAB3700672); FITC-labeled mouse anti-rat IgM antibody (clone MRM-47, Biolegend, catalog #408905)*;* bovine serum albumin (BSA) fraction-V (Sigma); normal goat serum (NGS, Sigma); Sigmacote^®^ (a siliconizing reagent, Sigma, catalog #SL-100 ml). Agar powder (catalog #A9539) and intralipid (20% v/v, catalog #I141) were form Sigma. Neutralizing anti-α5 integrin antibody (clone 5H10-27(MFR5), rat IgG2a, *k*, Biolegend, catalog #103817). Rat IgG2a, *k*, isotype control antibody (clone 2A3, IgG2a *k*, BioXcell, catalog #BE0089). Bioluminescent MB49-Luc murine bladder cancer cells were kindly provided by Carla Molthoff (VU University Medical Center, The Netherlands) and cultured as described [15]. Synthetic urine consisting of 128 mM sodium chloride, 60 mM potassium chloride, 40 mM sodium phosphate, 303 mM urea, 50 µg/ml bovine serum albumin and 2 mg/ml creatinine, pH 6.0, was prepared as described [19].

### Peptide synthesis and characterization

The head-to-tail cyclized peptide [CphgisoDGRG], called Iso4, was synthesized in-house. Briefly, the resin-bound linear precursor (CphgisoDGRG-resin) was assembled by standard stepwise solid-phase peptide synthesis (SPPS) protocols on a 2-chlorotrityl chloride resin using HBTU/DIEA as activators. The fully protected peptide was then detached from the resin by treatment with a 25% hexafluoropropanol solution in dichloromethane (4 × 5 mL). The solvent was removed under vacuum and the resulting crude linear peptide was dissolved in N,N-dimethylformamide (100 mM) and treated with HBTU/DIEA (1 eq./2 eq) to perform the cyclization step. The reaction was allowed to proceed overnight at room temperature and then the solvent was evaporated. The resulting product was then treated with a TFA-based cleavage mixture to obtain the unprotected peptide, which was recovered by precipitation in cold diethyl ether. Finally, the peptide was purified by reverse-phase (RP)-HPLC and lyophilized (final yield: about 100 mg as gross weight).

The peptide was dissolved in sterile water and stored in aliquots at − 80 °C until use. The concentration of Iso4 was determined by Ellman’s assay using 5,5-dithio-bis-2-nitrobenzoic acid (DTNB, Ellman’s Reagent, Thermo Fisher Catalog #22582). The identity and purity of Iso4 were assessed by mass spectrometry and HPLC analysis.

The percentage of [CphgisoDGRG] and of [CPhgisoDGRG] (where phg and Phg correspond to D-phenylglycine and L-phenylglycine, respectively) was estimated integrating the corresponding Hα resonances (respectively at 5.59 ppm and 5.55 ppm) in the ^1^H monodimensional nuclear magnetic resonance (NMR) spectrum. Spectra were acquired at 600 MHz on a Bruker Avance600 Ultra Shield Plus spectrometer equipped with a triple-resonance TCI cryoprobe with a z-shielded pulsed-field gradient coil. The following experimental conditions were used: 1 mM peptide in 20 mM sodium phosphate buffer, pH 6.5, containing 150 sodium chloride, 2.0 mM tris(2-carboxyethyl)phosphine (TCEP) and 10% D_2_O; temperature, 27 °C.

### Functionalization of gold nanorods with peptide Iso4

Eighty ml of GNRs in citrate buffer, pH 6.4 (with a longitudinal surface plasmon resonance (LSPR) peak maximum at ~ 820 nm and ~ 1 unit of optical density (OD)), were poured into a 150 ml silanized beaker, placed under stirring (500 rpm), and mixed with 8 ml of lipoic acid-polyethyleneglycol (PEG, 5KDa)-maleimide (1 mg/ml in 50 mM sodium phosphate buffer, pH 7.3, added dropwise over 2 min). The mixture was incubated at room temperature for 1 h under stirring, transferred into two silanized 50 ml polypropylene tubes, and centrifuged (9000 ×*g*, 45 min at 4 °C). The supernatants were discarded, and the pellets were resuspended with 5 mM sodium phosphate buffer, pH 7.3. The resulting products were pooled, transferred into a 20 ml silanized beaker, mixed with 10 ml of peptide Iso4 (0.160 mg/ml, by Ellman’s assay, in 5 mM sodium phosphate buffer, pH 7.3, added dropwise over 2 min, under stirring), and left to incubate for 2 h at room temperature. To saturate the gold nanorods we added 0.5% HSA (in 0.5 ml aliquots every 2 min, four times) and incubated for 10 min at room temperature under stirring. The product was then transferred to two 50 ml silanized polypropylene tubes and centrifuged as described above. Each pellet was resuspended in 0.05% HSA (40 ml) and centrifuged again (three rounds of centrifugation). The pellets were then resuspended in 8 ml of 0.05% HSA (final volume). The resulting product (called GNRs@PEG-Iso4) was dispensed in aliquots (0.5 ml) and stored at − 80 °C. Control nanoparticles bearing a cysteine in place of Iso4 (called GNRs@PEG-Cys) were prepared following the same procedure, except that 0.390 mg of cysteine was used in place of peptide.

### Physicochemical characterization of the functionalized nanoparticles

Absorption spectra of bare and functionalized GNRs (hereinafter called uncoated and coated, respectively) were recorded using an UltroSpec 2100 spectrophotometer (Amersham Biosciences). HSA (0.05% w/v) or 5 mM sodium citrate buffer, pH 6.0, respectively, were used as “blanks”. The concentration of coated-GNRs was calculated by interpolating the absorbance values at 820 nm on a calibration curve obtained using uncoated nanogold (stock solution: 4.3 × 10^11^ nanoparticles (NPs)/ml, λ_max_820 nm: ~ 1.0 OD, 23 µg/ml). Transmission electron microscopy (TEM) analysis was performed using a TALOS L120C microscope (ThermoScientific) and undiluted samples. Morphometric analysis of GNRs was performed on TEM images using the ImageJ software. Table 1 summarizes the physical properties of GNRs.

**Table 1:** Characterization of coated and uncoated gold nanorods (GNRs) by ultraviolet, visible, and infrared spectroscopy (UV–IR), transmission electron microscopy (TEM), and α5β1 integrin binding assay

Nanogold	UV–IR^a^ (*Mean* ± *SD)*				TEM (*Mean* ± *SD)*		α5β1 binding assay (*Mean* ± *SE)*
	TSPR (nm)	LSPR (nm)	PW-85% (nm)	A_510_/A_820_	Length (nm)	Width (nm)	EC_50_ (NPs/ml)
Uncoated GNRs	509 ± 1.1 *(n* = *4)*^b^	818 ± 2.3 *(n* = *4)*^b^	83.3 ± 1.3 *(n* = *4)*^b^	0.28 ± 0.01 *(n* = *4)*^b^	43.9 ± 5.4^e^	10.2 ± 0.7^e^	NA^h^
Uncoated GNRs in 5% NaCl	0.517	> 900^d^	> 100	0.59	ND^f^	ND	ND
*Coated GNRs*							
GNRs@PEG-Cys	511 ± 1.4 *(n* = *2)*^c^	827 ± 1.4 *(n* = *2)*^c^	99.8	0.33	41.0 ± 10.4 *(n* = *100)*^g^	12.9 ± 3.6 *(n* = *100)*^g^	> > 10^11 i^ *(n* = *3)*^c^
GNRs@PEG-Iso4	511 ± 1.0 *(n* = *3)*^c^	832 ± 2.0 *(n* = *3)*^c^	92.1 ± 3.4 *(n* = *3)*^c^	0.33 ± 0.02 *(n* = *3)*^c^	42.1 ± 9.4 *(n* = *100)*^g^	11.9 ± 3.3 *(n* = *100)*^g^	1.59 × 10^10^ ± 0.33 *(n* = *3)*^c^
GNRs@PEG-Iso4 in 5% NaCl	512	832	94.3	0.33	ND	ND	ND

^a^*TSPR*, transverse surface plasmon resonance (peak λ_max_); *LSPR*, longitudinal surface plasmon resonance (peak λ_max_); *PW-85%*, peak-width at 85% of height; A_510_/A_820_: absorbance ratio at 510 and 820 nm

^b^Number of independent analyses on the same batch

^c^Number of independent preparations analyzed

^d^Maximum wavelength that can be recorded by the UltroSpec spectrophotometer

^e^As reported on the datasheet provided by the supplier (Nanocompoxis)

^f^*ND*, not done

^g^Number of nanoparticles analyzed

^h^*NA*, not applicable

^i^Maximum concentration tested that gave no binding

### Stability of GNRs@PEG-Iso4 in synthetic urine

GNRs@PEG-Iso4 (0.1 ml, about 8 units of optical density at 820 nm) was added to synthetic urine (1 ml) and the resulting mixture was analyzed by spectrophotometry. Synthetic urine containing 0.05% HSA (i.e., the diluent of nanogold) was used as “blank” reference.

### α5β1 integrin binding assay

The binding properties of GNRs@PEG-Iso4 were investigated using a sandwich assay based on the use of α5β1-coated plates in the capture step and an anti-PEG monoclonal antibody (mAb) in the detection step, essentially as described [20]. Briefly various amounts of nanoparticles in 25 mM Tris–HCl buffer, pH 7.4, containing 150 mM sodium chloride, 1 mM magnesium chloride, 1 mM manganese chloride, 1% w/v BSA (*binding buffer*), were added to microtiter plates coated with or without human recombinant α5β1 (1–2 µg/ml, 50 µl/well) and incubated for 1.5 h. After washing, plates were incubated with a rat anti-PEG monoclonal antibody (clone 26A04) in *binding buffer* containing 1% v/v normal goat serum (NGS) (5 µg/ml, 50 µl/well, 1.5 h), followed by a goat anti-rat HRP-labelled polyclonal antibody (1:2000, 50 µl/well, 1 h). Bound peroxidase was detected by adding the chromogenic substrate o-phenylenediamine.

### FACS analysis

The expression of α5β1, αvβ3, αvβ5, and αvβ6 on MB49-Luc cell surface was assessed using of the monoclonal antibodies listed in Additional file 1: Table S2, as described previously [21]. Isotype-matched antibodies were used as negative controls. The binding of primary antibodies was detected using Alexa Fluor 488-labeled goat anti-mouse, or -hamster, or -rat secondary antibodies according to their animal species.The binding of GNR@PEG-Iso4 to MB49-Luc cells was assessed by flow cytometry analysis essentially as described previously [15]. Briefly, MB49-Luc cells were detached with DPBS containing 5 mM EDTA, pH 8.0, washed with DPBS, and suspended in 25 mM Hepes buffer, pH 7.4, containing 150 mM sodium chloride, 1 mM magnesium chloride, 1 mM manganese chloride_,_ 1% w/v BSA, 2% v/v NGS (*binding buffer-1*) and GNRs@PEG-Iso4 or GNRs@PEG-Cys (range 0–1 × 10^11^ NPs/ml, 5 × 10^5^ cells/100 μl tube). After 1 h incubation on ice, the cells were washed with *binding buffer-1* (without BSA and NGS) and resuspended in *binding buffer-1* containing the anti-PEG mAb 26A04 (1 µg/ml, 0.5 h on ice) followed by a FITC-labelled mouse anti-rat mAb MRM-47 (2.5 µg/ml, 0.5 h on ice). After washing, with DPBS/Ca/Mg, cells were fixed with 4% formaldehyde, and bound fluorescence was detected using a CytoFLEX S cytofluorimeter (Beckman Coulter).

### Orthotopic bladder cancer model

The tumor-binding properties of GNRs@PEG-Iso4 were investigated using an orthotopic mouse model of bladder cancer based on intravesical instillation of MB49-Luc cells. Briefly, female albino C57BL/6J mice (9 weeks old, weighing about 20 g, Charles River Laboratories, Italy) were anesthetized and intravesical instilled with MB49-Luc cells (10^5^ cells/100 µl in DPBS) using 24-gauge catheter. The tumor growth was monitored by ultrasound (US) imaging using a Vevo LAZR-X imaging system (FUJIFILM VisualSonics). After 11–14 days from tumor cells implantation mice were subjected to US and photoacoustic imaging (PAI) studies using a Vevo LAZR-X imaging system. All imaging experiments on mice were conducted under gaseous anesthesia (isoflurane/air 4% for induction and 1.5% thereafter).

### In vitro photoacoustic imaging

The in vitro photoacoustic properties of GNRs@PEG-Iso4 were investigated using two different homemade setups, the first one consisting in a coplanar net of polyethylene capillary tubes (Scientific Commodities Inc., cat. #BB31695-pe/8) inserted in a polypropylene box [22], the second one in "*agar drops*" (prepared as described in [15]) filled with various amounts of nanoparticles diluted in 0.05% HSA. The PA signal from the phantoms was then recorded using a Vevo LAZR-X imaging system with specially designed light attenuators, covering the optical fibers, made with a mixture of polydimethylsiloxane/TiO_2_/India ink [23] (for analysis of tube-based phantoms) or with a mixture of agar/intralipids [15] (for analysis of the agar drop-based phantoms). Ultrasound imaging of phantoms was performed in B-mode in the axial orientation (2D Power, 100% and 2D Gain, 13 dB). PA imaging of phantoms was performed in PA Mode Spectro (acquisition range; 680–970 nm; step size: 5 nm; PA Power, 100%; PA gain 43–45 dB), and in PA single wavelength mode (830 nm) for capillary tubes or in PA Mode 3D multi-wavelengths (3D step size: 200 µm) for agar drops. The signal corresponding to GNRs@PEG-Iso4 was identified by spectral unmixing using the signal derived from phantoms lacking nanoparticles, using the build-in VevoLab 5.6.1 software.

### In vivo photoacoustic imaging

In vivo photoacoustic imaging was performed as follows: GNRs@PEG-Iso4 (26 nmol Au in 100 µl DPBS/Ca/Mg) were instilled into the bladder of mice via a catheter and incubated for 15 min (every 5 min the bladder content was mixed with 3 cycles of aspiration/injection with a syringe connected to the catheter). The bladder content was then aspirated, and the excess of unbound nanoparticles was removed by washing the bladder twice with DPBS/Ca/Mg. US imaging of the bladder was performed using the transducer placed perpendicular to the mouse abdomen (B-mode: 2D Power, 50% and 2D Gain, 23 dB). PAI imaging of the bladder was performed essentially as described above (using agar/intralipids-based light attenuators), except that PA gain was set to 39 dB. PA analysis was performed by spectral unmixing using the spectral reference curves obtained from the tissue components (i.e., melanin and deoxygenated/oxygenated blood) and the GNRs@PEG-Iso4 spectral curve (generated as described above), using the build-in VevoLab 5.6.1 software.

## Results

### Characterization of peptide Iso4

**Fig. 1:**
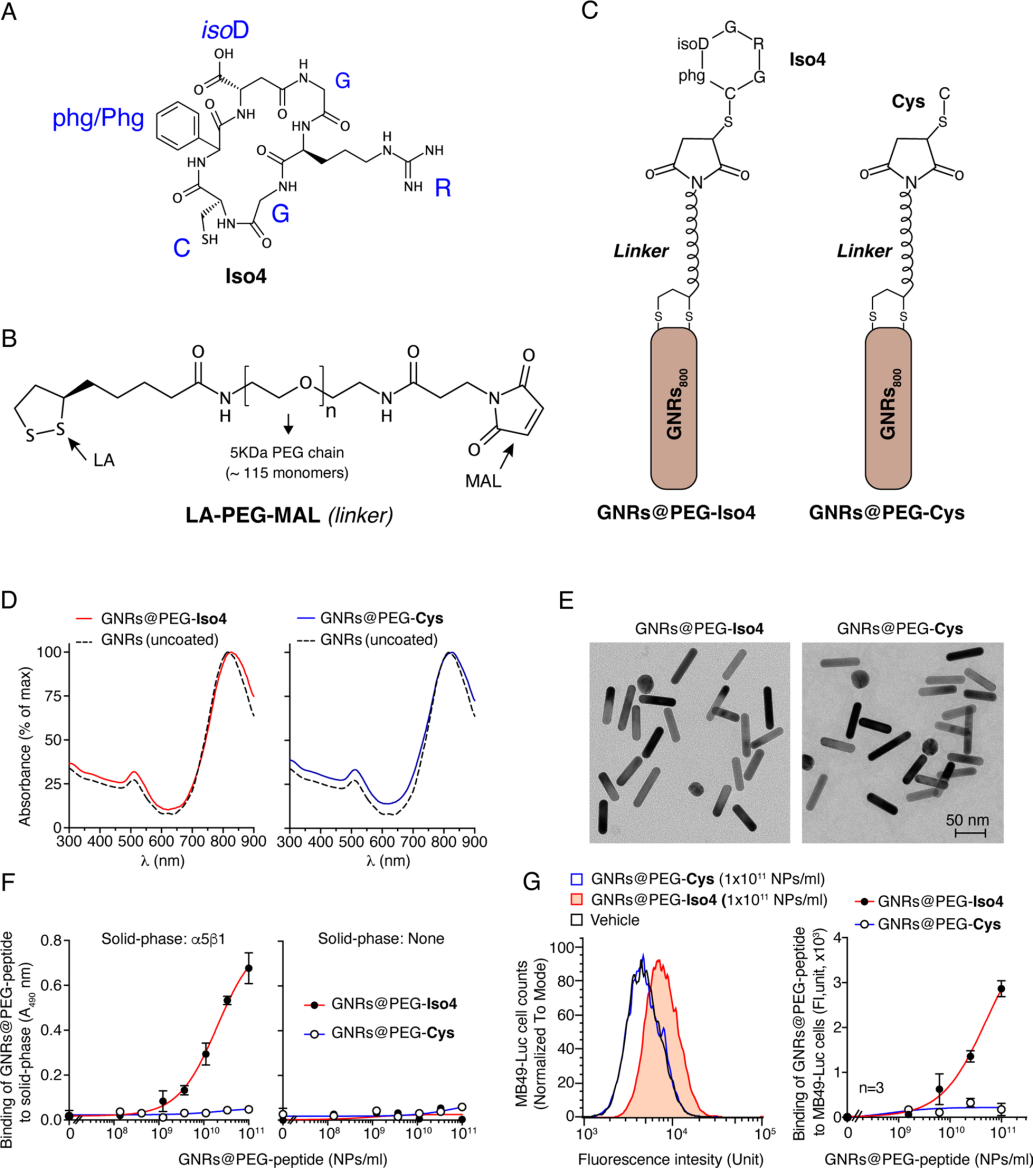
Schematic representation and characterization of GNRs@PEG-Iso4 and GNRs@PEG-Cys. Representation of the structures of: **A** head-to-tail cyclized peptide Iso4, **B** lipoic acid-PEG-maleimide heterobifunctional cross-linker (LA-PEG-MAL) containing a PEG chain of 5KDa, **C** and gold nanorods (GNRs) functionalized with peptide Iso4 (GNRs@PEG-Iso4) or Cys (GNRs@PEG-Cys) via LA-PEG-MAL. **D** UV-IR absorption spectra of the GNRs@PEG-Iso4 and GNRs@PEG-Cys. The dotted line corresponds to the uncoated gold nanorods (GNRs). **E** Representative microphotographs of GNRs@PEG-Iso4 and GNRs@PEG-Cys, as determined by transmission electron microscopy (TEM). **F** Binding of GNRs@PEG-Iso4 and GNRs@PEG-Cys to plates coated with or without α5β1-integrin. The binding of nanoparticles was detected with an anti-PEG rat antibody followed by HRP-labelled goat anti-rat antiserum. Mean ± SE of technical duplicates. **G** Binding of GNRs@PEG-Iso4 and GNRs@PEG-Cys to bladder cancer MB49-Luc cells as determined by FACS. MB49-Luc cells in suspension were incubated with the indicated amounts of nanoparticles for 1 h on ice. After washing, the cells were incubated with an anti-PEG antibody (0.5 h on ice) followed by a FITC-labelled secondary antibody (0.5 h in ice). The bound fluorescence was quantified by flow cytometry analysis. Representative FACS plots (*left*) and dose-dependent binding curves (*right*) (dots, mean ± SE of technical triplicates) are shown

Purity, identity and activity of the head-to-tail cyclized peptide Cys-phg-isoAsp-Arg-Gly ([CphgisoDGRG], called Iso4, Fig. 1A) were characterized by reverse-phase HPLC (RP-HPLC), electrospray ionization mass spectrometry (ESI–MS), nuclear magnetic resonance (NMR) spectroscopy, and integrin binding assays. RP-HPLC showed a purity > 95%; ESI–MS revealed a molecular weight consistent with the expected value; NMR spectroscopy showed the presence of D-phenylglycine (phg) (70%) and L-phenylglycine (Phg) (30%), as judged from the peak height of the resonances corresponding to the HαD and HαL of D-phenylglycine and L-phenylglycine (Additional file 1: Fig. S1 and Table S1); competitive integrin binding assays showed *Ki* values for integrin α5β1, αvβ6, αvβ8, αvβ5 and αvβ3 equivalent to 15, 46, 51, 1121, and 1493 nM, respectively (Additional file 1: Table S1). Direct integrin binding assays showed that Iso4 chemically coupled to maleimide-activated horseradish peroxide (HRP) can recognize α5β1 with a markedly higher affinity than αvβ3 and αvβ5, suggesting that coupling the peptide to HRP does not affect its selectivity (Additional file 1: Fig. S2).

### Preparation and characterization of GNRs@PEG-Iso4

GNRs@PEG-Iso4 were prepared by a two-step procedure. The first step included GNRs activation with lipoic acid-polyethylene glycol 5KDa-maleimide (LA-PEG-MAL), a heterobifunctional crosslinking reagent (Fig. 1B). The lipoic acid moiety of this reagent can react with the nanogold surface forming two stable dative bonds between the two sulfur atoms of the lipoic moiety and gold atoms [24]. The second step included GNRs functionalization with Iso4, by allowing the sulfhydryl group of the peptide to react with the maleimide group of the activated nanoparticles to form a thioether bond (Fig. 1C). Optimization studies showed that 90–120 µg of LA-PEG-MAL per ml of nanogolds (~ 1 OD_800nm_) was sufficient to protect nanogold from peptide-induced aggregation (not shown). Thus, a larger batch of GNRs@PEG-Iso4 was prepared using 80 ml of GNRs, 8 mg of LA-PEG-MAL, and 8 mg of Iso4 peptide. In parallel, control nanoparticles bearing cysteine in place of Iso4 were also prepared (GNRs@PEG-Cys).

UV-IR spectrophotometric analysis of both products showed absorption spectra similar to that of uncoated GNRs, indicating that both products contained low or undetectable amounts of aggregates (Fig. 1D and Table 1).

Morphometric analysis of GNRs@PEG-Iso4 and GNRs@PEG-Cys, by transmission electron microscopy (TEM), revealed, in both cases, gold nanorods with longitudinal and transversal length of 42 nm and 12 nm with an average aspect ratio (length/width) of about 3.53 (Fig. 1E and Table 1).

### GNRs@PEG-Iso4 binds to purified α5β1 integrin and α5β1-positive MB49-Luc bladder cancer cells in vitro

The capability of GNRs@PEG-Iso4 and GNRs@PEG-Cys to recognize integrin α5β1 was then investigated using recombinant α5β1-coated plates. The binding of nanoparticles to microtiter plates was detected with an anti-PEG mAb. The results showed that GNRs@PEG-Iso4, but not GNRs@PEG-Cys, could bind to α5β1 in a dose-dependent manner, with an effective concentration 50 (*EC*_*50*_) of (1.59 ± 0.33) × 10^10^ NPs/ml (Fig. 1F and Table 1).

To assess the capability of GNRs@PEG-Iso4 of recognizing bladder cancer cells we then analyzed the binding of these nanoparticles to MB49-Luc cells, a murine bladder cancer cell line that expresses α5β1 (Additional file 1: Fig. S3). Flow cytometry analysis of MB49-Luc cell suspensions, pre-incubated with various amounts of GNRs@PEG-Iso4, showed that these cells were recognized by nanoparticles in a dose-dependent manner, while little or no binding at all was observed with GNRs@PEG-Cys (Fig. 1G). TEM analysis of adherent cells pre-incubated with 1 × 10^11^ NPs/ml showed that GNRs@PEG-Iso4, but not GNRs@PEG-Cys could bind to the MB49-Luc cell surface (Additional file 1: Fig. S4).

This result suggests that GNRs@PEG-Iso4 can recognize the surface of bladder cancer cells, and that the Iso4 moiety is crucial for binding.

### Stability of GNRs@PEG-Iso4

**Fig. 2:**
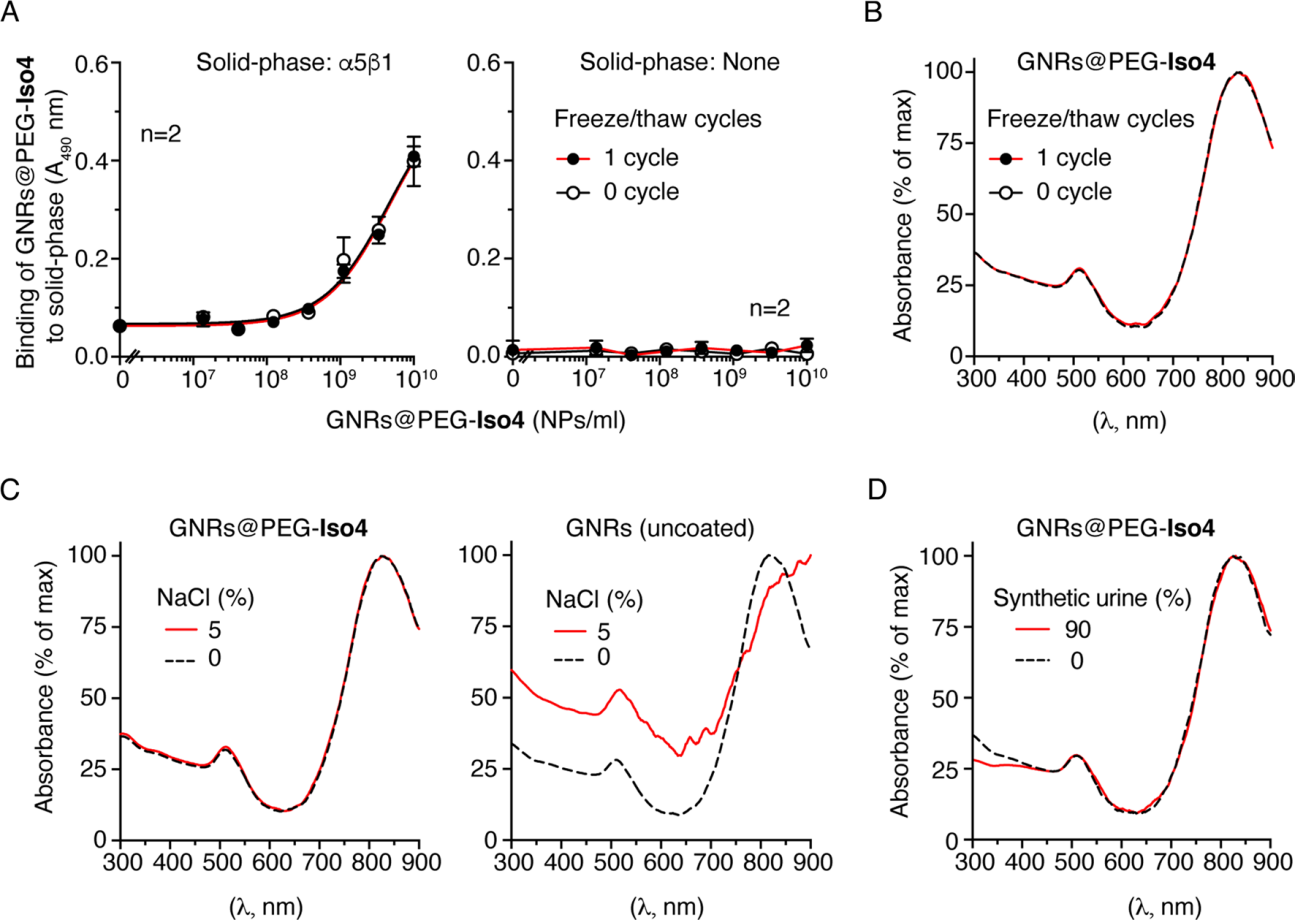
Stability studies of GNRs@PEG-Iso4. **A** Effect of one freeze–thaw cycle on the binding properties of GNRs@PEG-Iso4 to α5β1. The binding of GNRs@PEG-Iso4 to plates coated with or without α5β1 integrin before (0 cycle) and after one freezing (-80 °C) and thawing cycle (1 cycle), as detected with an anti-PEG rat antibody followed by HRP-labeled goat anti-rat antiserum, is shown. Mean ± SE of technical duplicates. Note that the freeze–thaw cycle does not affect the α5β1-binding properties of GNRs@PEG-Iso4. **B-D** Stability studies of GNRs@PEG-Iso4 and GNRs nanoparticles, performed by UV-IR absorption analysis of: *i*) nanoparticles before (*0 cycle*) and after one freezing (-80 °C) and thawing cycle (*1 cycle*) (**B**), *ii*) nanoparticles mixed with or without sodium chloride (5% NaCl, final concentration) (**C**), and *iii*) nanoparticles mixed with synthetic urine (90% urine, final concentration) (**D**). Note that the addition of sodium chloride caused a dramatic change in the UV-IR absorption spectrum of GNRs, but not of GNRs@PEG-Iso4, suggesting that the latter compound is protected from aggregation induced by high salt concentration

The binding of GNRs@PEG-Iso4 to α5β1 was not affected by one cycle of freezing and thawing (Fig. 2A). Moreover GNRs@PEG-Iso4 can be stored up to 4 months at 4 °C without loss of α5β1integrin binding properties, suggesting that this chemical bond is indeed very stable (Additional file 1: Fig. S5). To further assess the stability of GNRs@PEG-Iso4 to freezing/thawing- or salt-induced aggregation, this product was stored at + 4 °C and – 80 °C, or spiked with a large amount of sodium chloride (5% final concentration), and analyzed by UV-IR spectrophotometry. These treatments, known to induce aggregation of uncoated nanoparticles, did not change the absorption spectrum of GNRs@PEG-Iso4 (Fig. 2B, C), indicating that the nanoparticles were resistant to aggregation. Notably, incubation of GNRs@PEG-Iso4 in ~ 90% synthetic urine did not cause significant change in its absorption spectrum, suggesting that this product is stable also in urine (Fig. 2D**).**

To verify that GNRs functionalization with Iso4 did not affect the capability of GNRs@PEG-Iso4 to induce photothermal effects, we irradiated a cuvette containing the nanoparticles (500 µM) at 23 °C with continuous NIR light (808 nm) with a laser potency of 0.2, 0.3, and 0.4 W/cm^2^ for 15 min. This treatment increased the temperature of the cuvette to 37 °C, 44 °C, and 50 °C, respectively (Additional file 1: Fig. S6A). In contrast, none of the laser potency tested increased the solvent (0.05% HSA) temperature above 25 °C, indicating that nanoparticles were the source of the observed photothermal effects (Additional file 1: Fig. S6A). The percentage of light-to-heat conversion efficiency, calculated as previously described [25, 26] ranged from 58 to 70%, suggesting that more than 50% of the absorbed light can be converted in heat in our experimental conditions by GNRs@PEG-Iso4. Remarkably, no change of sample color and no aggregation of nanoparticles was observed at all temperatures, including 50 °C, indicating that GNRs@PEG-Iso4 was stable to heat (not shown). Moreover, after return to the baseline temperature, GNRs@PEG-Iso4 (125 µM) could generate the same amount of heat after an additional cycle of illumination, further supporting the concept that these nanoparticles are stable (Additional file 1: Fig. S6B). Overall, these results indicate that GNRs@PEG-Iso4 is a robust nanosystem.

### GNRs@PEG-Iso4 is endowed of photoacoustic properties

**Fig. 3:**
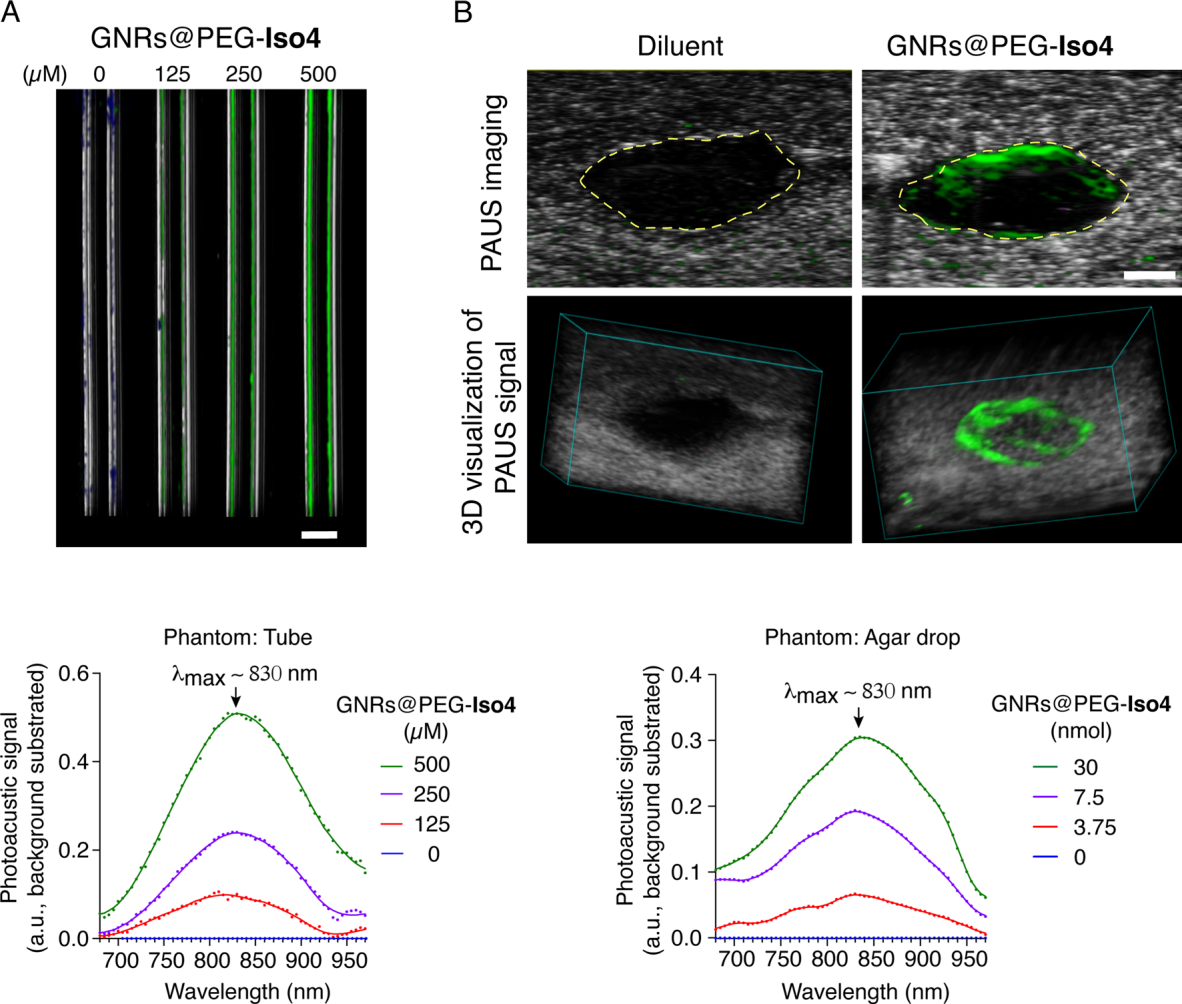
In vitro PA and US imaging of GNRs@PEG-Iso4. **A** Representative 2D PA and US image of capillary tubes filled with the indicated amount of GNRs@PEG-Iso4 (*upper*), and quantification of PA signal (*lower*). *Grayscale*, co-registered US signal; *Green*, PA signal of NPs. *Bar*, 1 mm. **B** Representative 2D and 3D PA and US images (*upper and middle panels, respectively)* of an agar drop with or without GNRs@PEG-Iso4 (30 µl nanogold, 30 nmol Au, ~ 1.16 × 10^11^ NPs), and quantification of PA signal (*lower*). The yellow *dotted line* in the *upper* marks the boundary between agar drop and the slime gel. *Grayscale*, co-registered US signal; *Green*: PA signal. *Bar*, 1 mm. Note that GNRs accumulated mainly at the periphery of the drop during the polymerization of the agar/GNRs mixture

The photoacoustic properties of GNRs@PEG-Iso4 were then investigated in vitro using a Vevo LAZR-X imaging system and two different types of phantoms, consisting of polyethylene capillary tubes and "*agar drops*" containing various amounts of GNRs@PEG-Iso4. The photoacoustic spectra of GNRs@PEG-Iso4 dispersed in these different phantoms were similar with a maximum signal at ~ 820–840 nm (Fig. 3), indicating that GNRs@PEG-Iso4 is endowed of photoacoustic properties. The spectral characteristics of GNRs@PEG-Iso4 obtained from the agar drop-based phantom (containing the higher number of NPs), was then used as a reference for the spectral unmixing in in vivo experiments.

### Intravesical instillation of GNRs@PEG-Iso4 to mice bearing orthotopic MB49-Luc bladder tumors allows photoacoustic imaging of small bladder cancer lesions

**Fig. 4:**
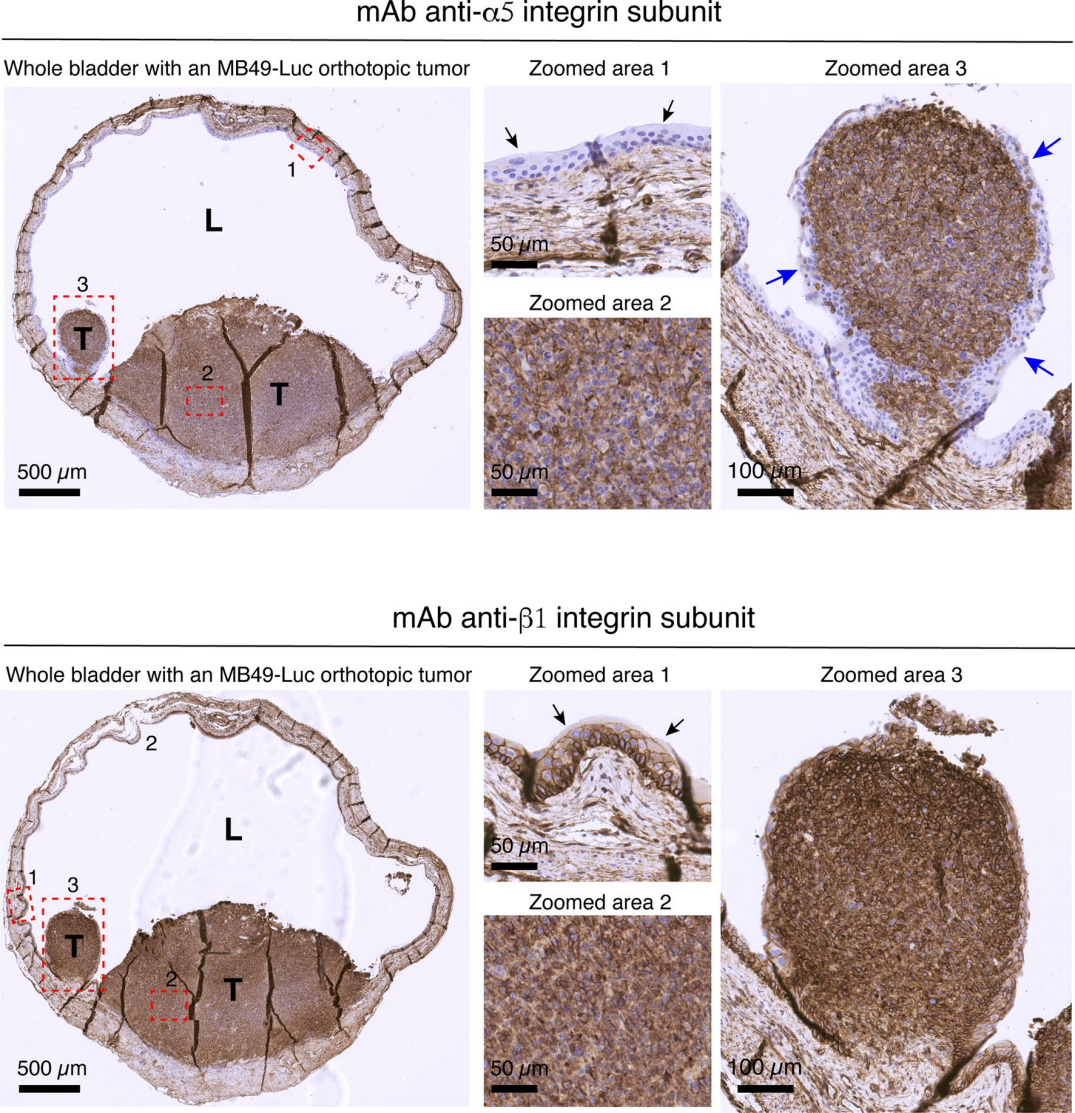
Expression of α5- and β1-integrin subunit in the MB49-Luc bladder cancer model. Representative immunohistochemistry photomicrographs of the expression of α5- and β1-integrin subunit in different areas of the bladder of a tumor-bearing mice, 11 days after intravesical instillation of MB49-Luc cells. This mouse developed two MB49-Luc tumors (T) with different size. *L*, lumen of the urinary bladder; *Red dashed rectangle*: zoomed areas. *Zoomed area 1*: healthy bladder, *black arrows* indicate the urothelial cells. *Zoomed area 2*: Inner MB49-Luc tumor. *Zoomed area 3:* Small tumor. The small tumor, but not the big one, is almost completely covered by 2–5 layers of α5-negative urothelial cells (*blue arrows).* Scale bar is shown in each panel. Immunostaining was performed as described previously [15]. *See* also Additional File 1: Fig. S7

The ability of GNRs@PEG-Iso4 to bind bladder cancer cells in vivo and to enable photoacoustic imaging of small cancer lesions was investigated using an orthotopic syngeneic model of bladder cancer based on murine MB49-Luc cells intravesical implanted in mice. In this model, neoplastic cells, but not non-neoplastic epithelial cells of the bladder, express α5β1 as determined by immunohistochemical analysis (Fig. 4 and Additional file 1: Fig. S7). Of note, this tumor model seems to grow under the urothelium and remains partially covered by some urothelial cell layers that show minimal or no α5β1 expression.

**Fig. 5:**
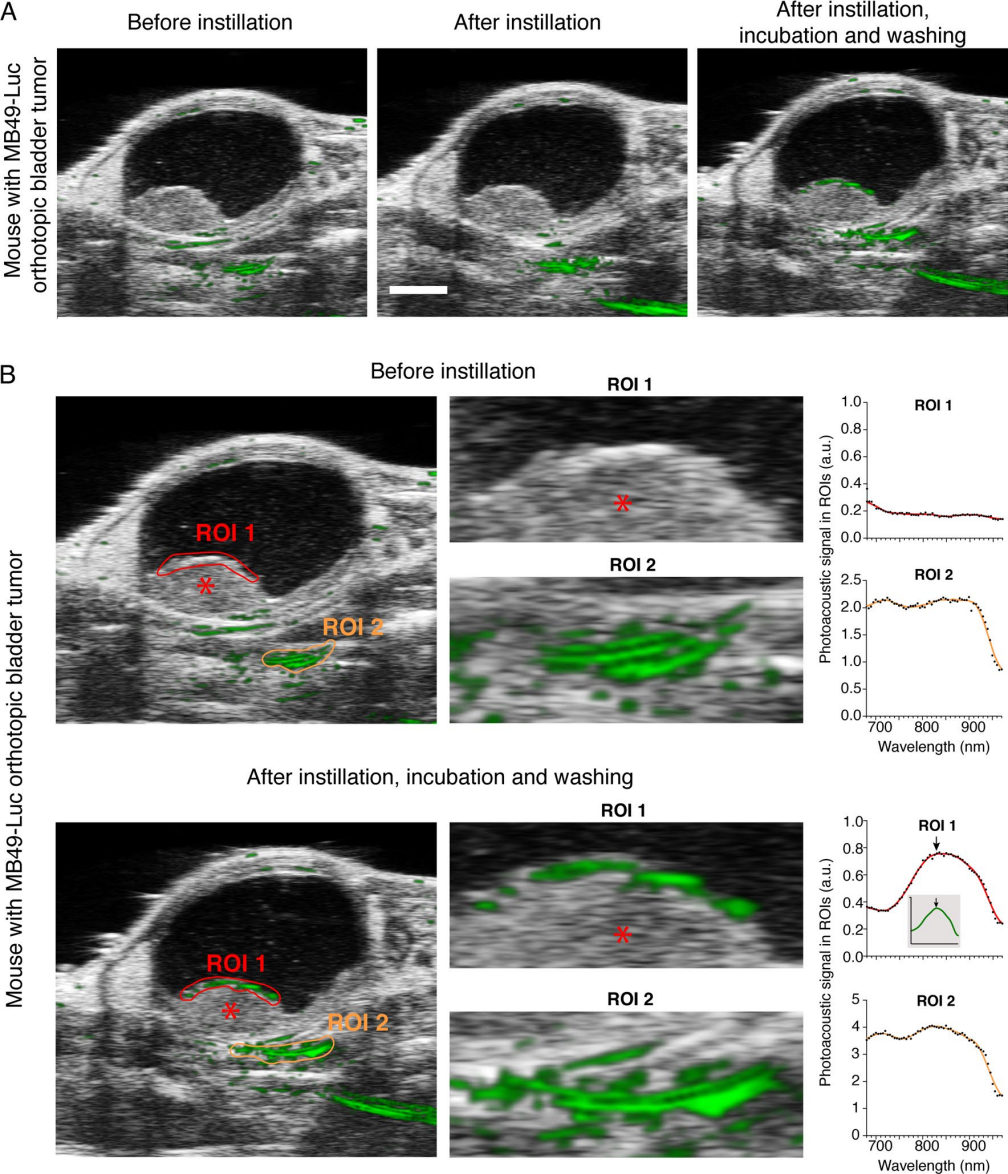
In vivo 2D PA and US images of an orthotopic MB49-Luc tumor before and after administration of GNRs@PEG-Iso4. MB49-Luc cells were implanted orthotopically in the bladder of a mouse. After 14 days, the bladder was imaged by PA and US analysis before and immediately after intravesical instillation of GNRs@PEG-Iso4 (26 nmol Au, ~ 1 × 10^11^ NPs), and after 15 min of incubation and bladder washing. **A** A representative axial 2D PA and US images of the entire bladder according to the indicated time of analysis. *Grayscale*, co-registered US signal; *Green*, PA signal *Bar*, 2 mm. **B** Zoomed images of *Panel A* and PA spectra in the selected ROIs according to the indicated time of analysis. *Asterisk*, Tumor; *Red* and *Orange* features delineate the ROIs drawn on the apical part of the tumor (ROI 1) and outside the bladder (ROI 2), respectively. Note that the PA spectrum detected in the tumor after installation of GNRs@PEG-Iso4, but not that of the PA signal observed outside the bladder, is very similar to the spectrum obtained with the same nanoparticles dispersed in agar drops (*inset*). *Arrows*, λmax: ~ 830 nm

To assess whether GNRs@PEG-Iso4 can accumulate on tumor lesions, we performed photoacoustic imaging studies on a mouse bearing MB49-Luc bladder cancer. Some scattered photoacoustic signals were observed in tissues outside the bladder, but not on tumor and bladder, before nanoparticle instillation (Fig. 5A, left panel), or immediately after GNRs@PEG-Iso4 administration (Fig. 5A, central panel). Specific photoacoustic signals were detected on the apical part of the tumor (i.e., on the luminal side of the bladder), after 15 min of incubation and bladder washing (Fig. 5A, right panel). Notably, the photoacoustic spectrum of the signals outside the bladder was different from that of GNRs@PEG-Iso4 (Fig. 5B), indicating that they were not related to gold nanoparticles. In contrast, the photoacoustic spectrum of the signal associated to the tumor showed a pattern very similar to that expected for gold nanoparticles (Fig. 5B), suggesting that, in this case, the signal was related to GNRs@PEG-Iso4 accumulation on the cancer lesion.

**Fig. 6:**
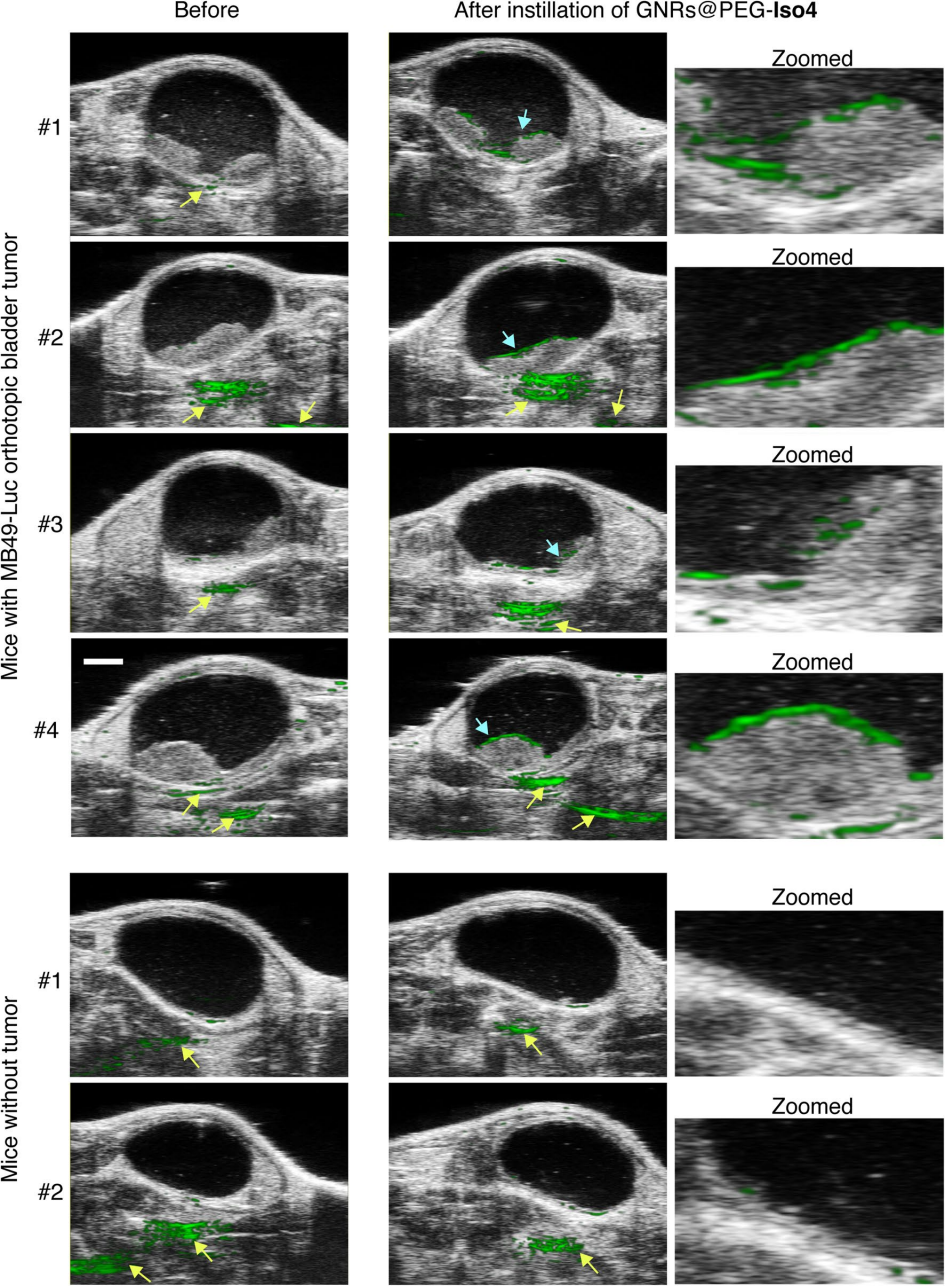
GNRs@PEG-Iso4 binds to orthotopic MB49-Luc bladder tumors but not to healthy bladder tissue. Representative axial 2D PA and US images of the bladder from tumor-bearing mice (n = 4 mice) or from healthy control mice (n = 2 mice) before and after the instillation of GNRs@PEG-Iso4 (26 nmol Au, ~ 1 × 10^11^ NPs), followed by incubation and bladder washing. Tumor-bearing mice were PA and US imaged 11–14 days after MB49-Luc cell implantation. *Grayscale*, co-registered US signal*; Green*, PA signal. *Cyan arrows*, PA signal generated by GNRs@PEG-Iso4. *Yellow arrows,* PA signal independent of GNRs@PEG-Iso4. *Bar*, 2 mm

**Fig. 7.**
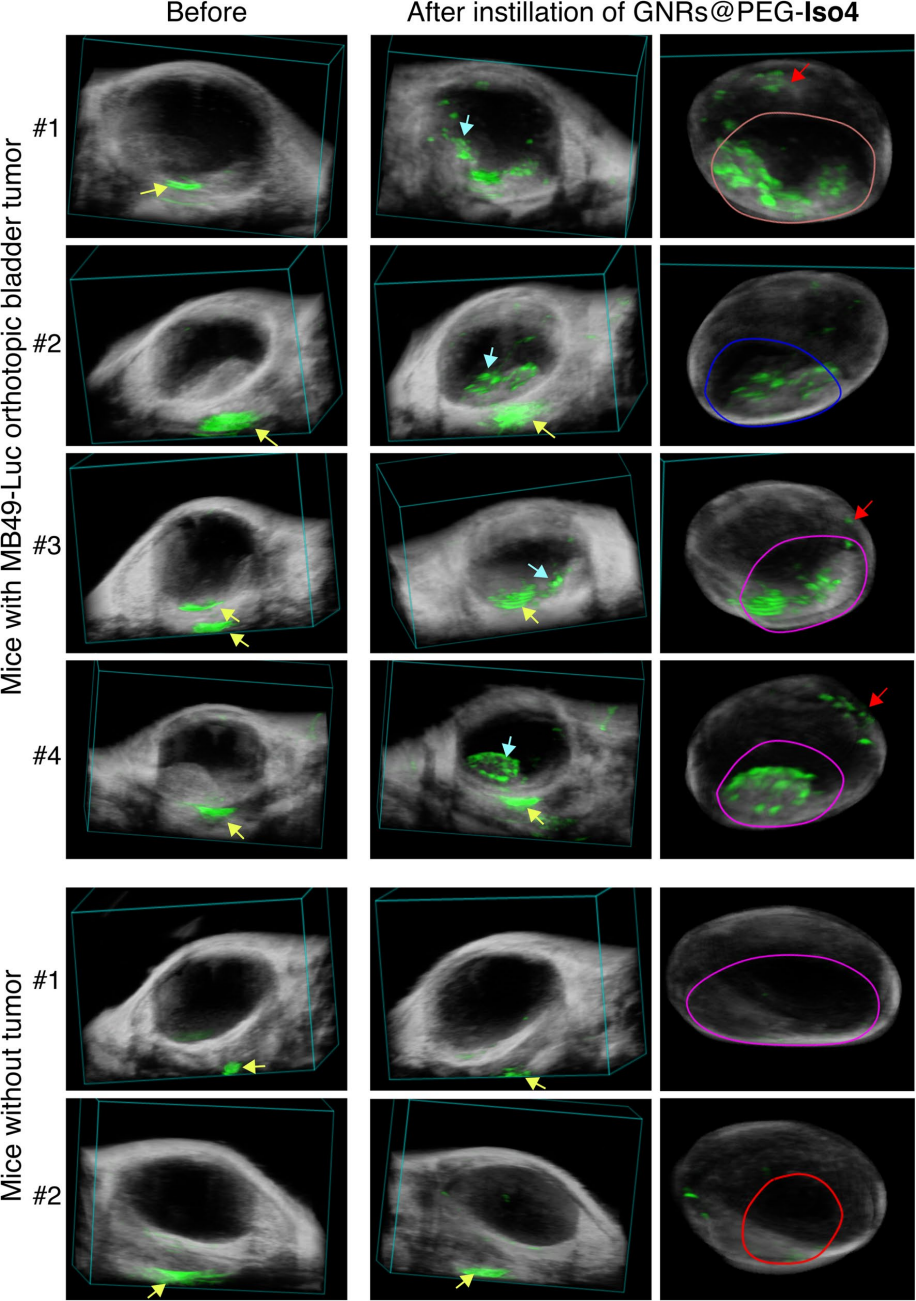
3D PA and US images of bladders from MB49-Luc tumor-bearing mice or healthy control mice before and after instillation of GNRs@PEG-Iso4. Representative 3D visualization (*left and middle panels*) and 3D reconstruction (*right panels*) of the PA and US signal of the bladders of mice of Fig. 6. *Grayscale*, co-registered ultrasound signal; *Green*, PA signal. *Cyan arrows*, specific signal of GNRs@PEG-Iso4. *Yellow arrows*, nonspecific signal recorded outside the bladder present before administration of GNRs@PEG-Iso4. Note the almost complete absence of PA signal inside the bladder in control animals. *Red arrows* indicate small spots of PA signal likely corresponding to small bladder cancer lesions (< 0.5 mm) that are undetectable by standard US echography

We next investigated the specificity and the capability of GNRs@PEG-Iso4 to detect tumor lesions of different size. To this aim GNRs@PEG-Iso4 was administered in 3 additional tumor-bearing mice and in 2 healthy mice. The result showed that GNRs@PEG-Iso4 could recognize tumor lesions in all tumor-bearing mice, but not the adjacent normal bladder tissue (Figs. 6 and 7, *upper panels*). No binding of GNRs@PEG-Iso4 to the bladder of healthy mice was observed (Figs. 6 and 7, *lower panels*). As expected, GNRs@PEG-Cys (a non-targeted nanoformulation having a cysteine residue in place of the Iso4 peptide) failed to detect tumor lesions, suggesting that peptide Iso4 is crucial for tumor recognition by nanoparticles (Additional file 1: Fig. S8).

Taken together these results suggest that GNRs@PEG-Iso4 can selectively recognize bladder cancer cells in vivo, thereby enabling the photoacoustic imaging of small cancer lesions.

### Role of α5β1 integrin as a target receptor of GNRs@PEG-Iso4 in MB49-Luc bladder tumors

To investigate the role of α5β1 on the binding of GNRs@PEG-Iso4, mice bearing orthotopic MB49-Luc tumors were intravesical injected with an α5β1-blocking antibody, or with an isotype-matched control antibody, followed, 15 min later by GNRs@PEG-Iso4. The bladders were washed again and the uptake of GNRs@PEG-Iso4 was quantified by photoacoustic imaging. A reduced photoacoustic signal was observed in mice treated with the α5β1-blocking antibody, suggesting that α5β1 is an important receptor for GNRs@PEG-Iso4 (Additional file 1: Fig. S9).

## Discussion

This work demonstrates that GNRs@PEG-Iso4 represents a stable and robust nanosystem for photoacoustic imaging of small α5β1-positive bladder cancer lesions.

GNRs@PEG-Iso4 consists of PEGylated gold nanorods that absorb light in the near-infrared region of the electromagnetic spectrum (peak maximum at ~ 820 nm), functionalized with the cyclic peptide [CphgisoDGRG] (Iso4), a ligand of α5β1 integrin. Notably, these nanoparticles can be prepared by a simple two-step procedure. In the first step, GNRs are activated with a heterobifunctional reagent consisting of (a) lipoic acid (which can form stable sulfur–gold bonds with the nanoparticle surface), (b) a PEG 5 kDa linker, and (c) a maleimide group (which can react with the thiol group of Iso4). In the second step, maleimide activated-GNRs are coupled to Iso4 via its thiol group (see Fig. 1). The results of biochemical and biological studies on these nanoparticles show that Iso4 preserves its functional properties after coupling to GNRs, as suggested by the observation that GNRs@PEG-Iso4 could specifically bind purified α5β1 and α5β1-positive bladder cancer cells (MB49-Luc). Furthermore, the results of stability studies show that GNRs@PEG-Iso4 is stable and does not aggregate in urine or in 5% sodium chloride, and even after one freeze/thaw cycle. Notably, the photothermal properties of GNRs@PEG-Iso4 were not affected after two cycles of on/off NIR laser illumination (which can raise the temperature up to 55 °C), suggesting a good thermal stability of GNRs@PEG-Iso4.

The results of in vivo photoacoustic imaging studies, performed in mice bearing orthotopic MB49-Luc bladder tumors, show that GNRs@PEG-Iso4 can bind the surface of well-established cancer lesions (α5β1-positive, detectable by standard US echography), but not to the bladder of heathy mice (α5β1-negative). Notably, small spots of photoacoustic signals were detected also at sites in which standard US echography failed to detect tumor lesions: considering that no uptake of nanoparticles was observed by the urothelium in healthy mice, these sites likely correspond to small bladder cancer lesions < 0.5 mm, undetectable by standard US echography. At variance, the photoacoustic signals observed in tissues around the bladder of both tumor-bearing and normal mice were likely related to artifacts dependent on the acoustic inhomogeneity of the tissue, which may cause signal reflection inside the imaging plane [27–29]. This hypothesis is supported by the observation that a) these signals were present also before nanoparticle administration, and b) these signals exhibited a photoacoustic spectrum completely different from that of GNRs@PEG-Iso4.

The tumor-binding properties of GNRs@PEG-Iso4 depend on a targeting mechanism mediated by α5β1-integrin expressed by the tumor cells, as suggested by the observation that (a) no binding occurred to α5β1-negative healthy bladder and (b) nanoparticle accumulation on tumor lesions was partially inhibited by pre-administration of a neutralizing anti-α5β1 monoclonal antibody. However, because the inhibition was not complete, we cannot exclude the possibility that other receptors are involved. Notably, immunohistochemical studies performed by other investigators have shown that healthy bladder tissues of mice and humans can express high levels of αvβ6 and αvβ8 [30, 31], i.e., two potential additional receptors of peptide Iso4 (*Ki* for α5β1, αvβ6 and αvβ8: 15, 46 and 51 nm, respectively). However, no binding of GNRs@PEG-Iso4 to the healthy mouse bladder was detected, suggesting that these receptors, at least those exposed to the luminal side, are somehow not "*accessible*" to GNRs@PEG-Iso4, possibly because they are already engaged by other ECM ligands or because they have a conformation corresponding to the inactive “resting” state of the integrin [32]. The photoacoustic signal of GNRs@PEG-Iso4, detected in MB49-Luc bladder cancer model, was characterized by a "*patchy pattern*" that was particularly evident in reconstructed 3D photoacoustic images. This peculiar pattern may be related to (a) heterogeneous expression of α5β1 molecules characterized by differential accessibility of the ligand [33], and (b) presence of discontinuous urothelial cell layers (α5β1-negative) above the tumor, which may prevent or reduce the binding of nanoparticles to the underlying tumor cells (α5β1-positive). At this regard, it is important to highlight the fact that the immunohistochemical characterization of in situ carcinomas-tissue sections obtained from patients showed that α5β1-positive cells are exposed to the bladder lumen, which are, therefore, accessible to intravesically administered nanoparticles [15]. Intravesical administration of NPs may represent an important advantage comparted to intravenous administration as local delivery may reduce potential systemic toxicological effects.

In summary, the results of PAI studies performed in the MB49-Luc model show that GNRs@PEG-Iso4 can specifically accumulate on α5β1-positive tumors, but not on the normal urothelium.

Other investigators have exploited the mucoadhesive properties of chitosan towards urothelial cells for the preparation of drug delivery systems that target bladder cancer [34–36]. Recently, we have reported that GNRs coated with chitosan and Iso4 (GNRs@Chit-Iso4) and instilled in the bladder of mice bearing MB49-Luc tumors, allows photoacoustic imaging of small cancer lesions (< 0.5 mm) undetectable by US, as confirmed by the results of a longitudinal study showing that the small areas recognized by these nanoparticles become detectable also by US 5 days later upon tumor growth [15]. Because GNRs@Chit-Iso4 particles tended to sediment at the bottom of the bladder after intravesical instillation, cancer imaging in tumor-bearing mice required the application of low-frequency US cycles with a piezoelectric matrix array transducer placed on the abdomen to enable nanoparticle distribution on the entire bladder surface [15], a procedure that may be difficult to translate in patients. Notably, the fact that GNRs@PEG5K-Iso4 does not undergo sedimentation after intravesical administration represents a major advantage over GNRs@Chit-Iso4, because in this case the use the piezoelectric matrix array transducer is not necessary. Furthermore, the fact that GNRs@PEG5K-Iso4 could still image bladder cancer lesions despite the lack of chitosan indicate that this compound is not crucial for the targeting mechanism of GNRs to tumors and suggest that nanoparticle functionalization with Iso4 is sufficient for tumor recognition. Consequently, another inherent advantage of GNRs@PEG5K-Iso4 is related to its simpler production procedure. Indeed, while GNRs@Chit-Iso4 preparation requires several reagents for Iso4 coupling (thioglycolic acid, ethyl(dimethylaminopropyl)carbodiimide, N-hydroxysuccinimide ester, chitosan, maleimide-PEG_12_-N-hydroxysuccinimide ester), GNRs@PEG-Iso4 requires only the LPA-PEG-MAL cross-linking reagent and a simpler coupling procedure, thus reducing the risk of nanoparticle heterogeneity. Thus, to summarize, the main differences between GNRs@Chit-Iso4 and GNRs@PEG-Iso4 are related to the size of GNRs (~ 90 nm length and ~ 25 nm width versus ~ 42 nm length and ~ 12 nm width, respectively), type of coating material (chitosan, a polysaccharide with an undefined molecular weight ranging from 50 to 200 kDa, versus PEG5KDa, an almost homogeneous compound with a polydispersity of 1.09), and the chemistry used for their assembly (4 distinct conjugation steps for GNRs@Chit-Iso4 and only 2 conjugation steps for GNRs@PEG-Iso4).

GNRs@PEG-Iso4 may have different applications in bladder cancer patients, such as diagnostic imaging and image-guided surgery of small lesions. Considering the photothermal effects observed after illumination with continuous laser light, GNRs@PEG-Iso4 might also be exploited for photothermal therapy of bladder cancer, a therapeutic application that deserves to be investigated. In addition, considering that α5β1 is expressed also by other tumor types, e.g. gastric tumors, endometrial cancer [37, 38], bone metastases of breast cancer, [39] and by the neovasculature of several solid tumors [32], the present results may stimulate other studies aimed at assessing the utility of GNRs@PEG-Iso4 for imaging and therapy of other tumors. Although no adverse reactions were observed during the imaging procedure, it is necessary to conduct adequate biosafety investigations on GNRs@PEG-Iso4 prior any clinical applications as a diagnostic and/or theranostic agent.

## Conclusion

GNRs@PEG-Iso4 represents a simple, homogeneous, and robust diagnostic tool for photoacoustic imaging and diagnosis of small bladder cancer lesions with the potential to be rapidly translated in the clinic.

### Supplementary Information

**Additional file 1: Table S1.** Biochemical characterization of peptide Iso4 by Ellman’s assay, electrospray ionization mass spectrometry (ESI–MS) analysis, RP-HPLC, nuclear magnetic resonance (NMR) spectroscopy and integrin binding. **Table S2.** Expression of cell surface integrins on murine MB49-Luc bladder carcinoma cells as determined by FACS analysis. **Figure S1.** Zoom into the ^1^H-1D NMR spectrum of peptide Iso4 centered on the Hα resonances of phg and Phg. NMR spectroscopy shows that peptide Iso4 consists of two isomers corresponding to a cyclic head-to-tail peptide with D-phenylglycine (phg) and L-phenylglycine (Phg), the latter accounting for about 30%. The HαD and HαL signals of phenylglycine (each giving rise to a doublet) are indicated. **Figure S2.** Binding of Iso4-HRP conjugate to microtiter plates coated with or without α5β1, αvβ3 and αvβ5. **A** Schematic representation of head-to-tail cyclized Iso4 and control peptides (Iso3 and ARA, positive and negative control, respectively). **B** Binding of peptide-HRP conjugates to microtiter plates coated with α5β1, αvβ3 and αvβ5. Peptide-HRP conjugates were prepared by coupling peptides to maleimide-activated horseradish peroxidase (HRP), as described in Ref. [15]. The binding assay was performed as described previously [15] using the indicated amount of integrins for microtiter plate coating. Mean ± SE, n = 2 wells. The Iso3-HRP and ARA-HRP were used as positive and negative controls, respectively, to assess integrin functionality (Iso3-HRP) and binding specificity (ARA-HRP). The binding curves of Iso4-HRP and ARA-HRP α5β1 are reprinted with the permission of Ref. [15]. **Figure S3.** Expression of α5-, β1-, β3-, β5-, αv-integrin subunits, and αvβ6 integrin on murine bladder MB49-Luc carcinoma cells. Integrin expression by MB49-Luc cells was analyzed by FACS using the indicated anti-integrin antibodies (5 µg/ml), and appropriate species-specific Alexa Fluor 488-labeled secondary antibodies (5 µg/ml). Binding of an isotype control antibody is also shown (see Table S2 for antibody description). **Figure S4.** TEM analysis of MB49-Luc cells incubated with GNRs@PEG-Iso4 or GNRs@PEG-Cys. MB49-Luc cells, cultured in a 12-well plate (cell confluency > 90%), were washed twice with 0.9% sodium chloride and then incubated for 5 min with 25 mM Hepes buffer, pH 7.4, containing 150 mM sodium chloride, 1 mM magnesium chloride, 1 mM manganese chloride, 1% w/v BSA. The cells were then incubated for 2 h at 37 °C, 5% CO_2_, with GNRs@PEG-Iso4 or GNRs@PEG-Cys (1 × 10^11^ NPs/ml, 500 μl well). The cells were then washed with the same buffer (3 times, 5 min each), fixed, and analyzed by TEM as described in Ref. S1. **Figure S5.** Characterization of GNRs@PEG-Iso4 stability by α5β1-integrin binding assay. Binding of GNRs@PEG-Iso4 (different lots*,* called *#A* and *#B*, corresponding to freshly prepared NPs or after 4 months of storage at 4 °C, respectively), to microtiter plated coated with or without α5β1. NPs binding was detected as described in *Methods*. GNRs@PEG-Cys was also included as a negative control. Note that the binding of GNRs@PEG-Iso4 Lot#B is similar to that of Lot#A, suggesting that no chemical detachment or degradation of the various compounds composing the NPs occurred during the storage for 4 months at 4 °C. **Figure S6.** Photothermal properties of GNRs@PEG-Iso4. The capability of GNRs@PEG-Iso4 to release heat after illumination (0–15 min) was tested using a custom-made system consisting of: *1*) a continuous-wave NIR laser line at 808 nm (LDC220C, TED200C, Thorlabs, laser power, 0.2, 0.3 and 0.4 W), *2)* a multimodal optical fiber (1 mm Ø, Thorlabs), *3*) a power meter sensor (Thorlabs, cat. s405c); *4*) a cuvette holder (Thorlabs), *5*) a polystyrene cuvette (Kartell, cat. 01938–00) containing 500 µl of sample, (0–500 µM of Au, final concentration) and *6*) a NIR camera (HEIMANN Sensor, cat. HTPA80 × 64dR2L3.9/0.8HiA). **A** Heating curve of GNRs@PEG-Iso4 (500 µM Au) dispersed in 0.05% HSA (*Diluent*) and irradiated with an 808 nm laser at the indicated power densities. *Right panel inset*, the image of the cuvette containing GNRs@PEG-Iso4 after 15 min of laser illumination at 0.4 W is shown. **B** Thermal stability of GNRs@PEG-Iso4 (125 µM Au) over 2 cycles of a laser on/off experiment at the indicated laser power. The 2nd cycle of a laser illumination was performed after 24 h from the 1st cycle. **Figure S7.** Expression of α5- and β1-integrin subunit in the MB49-Luc bladder cancer model. Representative immunohistochemistry photomicrographs of the expression of α5- and β1-integrin subunit in different areas of the bladder of a tumor-bearing mice, 15 days after intravesical instillation of MB49-Luc cells. *T*, MB49-Luc tumor; *L*, lumen of the urinary bladder; *Red dashed rectangle*: zoomed areas. *Zoomed area 1*: healthy bladder, *black arrows* indicate the urothelial cells. *Zoomed area 2*: Luminal side of MB49-Luc tumor. Note that this big tumor is minimally covered by layers of α5-negative urothelial cells (*blue arrows)*. Immunostaining was performed as described previously [15]. Adapted and reprinted with the permission of Ref. [15]. **Figure S8.** GNRs@PEG-Cys does not bind to orthotopic MB49-Luc tumor lesions. **A** Schematic representation of the experimental procedure. A mouse with orthotopic MB49-Luc tumor lesions underwent PAUS imaging of the bladder before (*Step 1*) and after (*Step 2)* intravesical instillation of GNRs@PEG-Cys (26 nmol Au). After bladder washing, additional PAUS imaging of the bladder was then performed before (*Step 3*) and after (*Step 4*) instillation of GNRs@PEG-Iso4 (26 nmol Au). **B** Representative PAUS images (axial 2D) of the bladder after step 1, 2, 3, and 4. *Grayscale*, co-registered US signal; *Green*, PA signal; *Cyan arrows*, specific PA signal (generated by GNRs@PEG-Iso4). *Yellow arrows*, unspecific PA signal (independent from nanoparticles). *Bar*, 2 mm. Specific PA signal on tumor lesions was observed only after step 4, suggesting that peptide Iso4 is crucial for tumor recognition by nanoparticles. **Figure S9.** Effect of a neutralizing anti-α5β1 mAb on the uptake of GNRs@PEG-Iso4 to MB49-Luc bladder tumors. Mice bearing orthotopic MB49-Luc tumors were intravescically administered with a control isotype mAb or a neutralizing anti-α5β1 mAb (clone: RTK2758 and 5H10-27(MFR5), respectively, 20 µg/mouse). After 15 min, the bladders were emptied and subsequently filled with GNRs@PEG-Iso4 (26 nmol Au in 100 µl, ~ 1 × 10^11^ NPs). After 15 min, the bladders were washed, and PA and US imaged. PA signals associated with whole tumors or adjacent healthy tissues (*background*) were quantified using VevoLab 5.6.1 software. Box-plots with median, interquartile and 5–95 percentile, in which dots represent well-established tumors. N = 4–5 mice, with one or two tumors per bladder. *P*, by Mann–Whitney nonparametric test.

## Data Availability

All data generated or analyzed during this study are included in this published article and its supplementary information files. Further information and requests for resources should be directed to Flavio Curnis (curnis.flavio@hsr.it) or Massimo Alfano (alfano.massimo@hsr.it).

## Abbreviations

GNRs: Gold nanorods
NPs: Nanoparticles
Iso4: Cyclic [CphgisoDGRG] peptide
NMR: Nuclear magnetic resonance
NIR: Near-infrared
PA: Photoacoustic
US: Ultrasound
PAI: Photoacoustic imaging

## References

[1] Ferlay J, Soerjomataram I, Dikshit R, Eser S, Mathers C, Rebelo M (2015). Cancer incidence and mortality worldwide: sources, methods and major patterns in GLOBOCAN 2012. Int J Cancer.

[2] Burger M, Catto JW, Dalbagni G, Grossman HB, Herr H, Karakiewicz P (2013). Epidemiology and risk factors of urothelial bladder cancer. Eur Urol.

[3] https://uroweb.org/guidelines/non-muscle-invasive-bladder-cancer. Accessed 10 Aug 2023.

[4] Ozden E, Turgut AT, Turkolmez K, Resorlu B, Safak M (2007). Effect of bladder carcinoma location on detection rates by ultrasonography and computed tomography. Urology.

[5] Babjuk M, Burger M, Comperat EM, Gontero P, Mostafid AH, Palou J (2019). European association of urology guidelines on non-muscle-invasive bladder cancer (TaT1 and carcinoma in situ)—2019 update. Eur Urol.

[6] van Rhijn BW, Burger M, Lotan Y, Solsona E, Stief CG, Sylvester RJ (2009). Recurrence and progression of disease in non-muscle-invasive bladder cancer: from epidemiology to treatment strategy. Eur Urol.

[7] Zapała P, Dybowski B, Poletajew S, Białek Ł, Niewczas A, Radziszewski P (2018). Clinical rationale and safety of restaging transurethral resection in indication-stratified patients with high-risk non-muscle-invasive bladder cancer. World J Surg Oncol.

[8] Leal J, Luengo-Fernandez R, Sullivan R, Witjes JA (2016). Economic burden of bladder cancer across the European Union. Eur Urol.

[9] Mariotto AB, Yabroff KR, Shao Y, Feuer EJ, Brown ML (2011). Projections of the cost of cancer care in the United States: 2010–2020. J Natl Cancer Inst.

[10] Szlachcic A, Pala K, Zakrzewska M, Jakimowicz P, Wiedlocha A, Otlewski J (2012). FGF1-gold nanoparticle conjugates targeting FGFR efficiently decrease cell viability upon NIR irradiation. Int J Nanomedicine.

[11] Cho SK, Emoto K, Su LJ, Yang X, Flaig TW, Park W (2014). Functionalized gold nanorods for thermal ablation treatment of bladder cancer. J Biomed Nanotechnol.

[12] Yang X, Su L-J, Rosa FGL, Smith EE, Schlaepfer IR, Cho SK (2017). The antineoplastic activity of photothermal ablative therapy with targeted gold nanorods in an orthotopic urinary bladder cancer model. Bladder Cancer.

[13] Chen CH, Wu YJ, Chen JJ (2016). Photo-thermal therapy of bladder cancer with anti-EGFR antibody conjugated gold nanoparticles. Front Biosci (Landmark Ed).

[14] Cho SK, Su LJ, Mao C, Wolenski CD, Flaig TW, Park W (2019). Multifunctional nanoclusters of NaYF(4):Yb(3+), Er(3+) upconversion nanoparticle and gold nanorod for simultaneous imaging and targeted chemotherapy of bladder cancer. Mater Sci Eng C Mater Biol Appl.

[15] Alchera E, Monieri M, Maturi M, Locatelli I, Locatelli E, Tortorella S (2022). Early diagnosis of bladder cancer by photoacoustic imaging of tumor-targeted gold nanorods. Photoacoustics.

[16] Wang R, Du N, Jin L, Chen W, Ma Z, Zhang T (2022). Hyaluronic acid modified Au@SiO2@Au nanoparticles for photothermal therapy of genitourinary tumors. Polymers-basel.

[17] Liao MY, Huang TC, Chin YC, Cheng TY, Lin GM (2022). Surfactant-free green synthesis of Au@Chlorophyll nanorods for NIR PDT-elicited CDT in bladder cancer therapy. ACS Appl Bio Mater.

[18] Curnis F, Sacchi A, Longhi R, Colombo B, Gasparri A, Corti A (2013). IsoDGR-tagged albumin: a new avb3 selective carrier for nanodrug delivery to tumors. Small.

[19] Shmaefsky B (1995). Artificial urine for laboratory testing: revisited. Am Biol Teach.

[20] Corti A, Sacchi A, Gasparri AM, Monieri M, Anderluzzi G, Colombo B (2021). Enhancement of doxorubicin anti-cancer activity by vascular targeting using IsoDGR/cytokine-coated nanogold. J Nanobiotechnology.

[21] Nardelli F, Ghitti M, Quilici G, Gori A, Luo Q, Berardi A (2019). A stapled chromogranin A-derived peptide is a potent dual ligand for integrins alphavbeta6 and alphavbeta8. Chem Commun (Camb).

[22] Armanetti P, Chilla A, Margheri F, Biagioni A, Menichetti L, Margheri G (2021). Enhanced antitumoral activity and photoacoustic imaging properties of AuNP-enriched endothelial colony forming cells on melanoma. Adv Sci (Weinh).

[23] Avigo C, Di Lascio N, Armanetti P, Kusmic C, Cavigli L, Ratto F (2015). Organosilicon phantom for photoacoustic imaging. J Biomed Opt.

[24] Sperling RA, Parak WJ (1915). Surface modification, functionalization and bioconjugation of colloidal inorganic nanoparticles. Philos Trans A Math Phys Eng Sci.

[25] Pasciak A, Pilch-Wrobel A, Marciniak L, Schuck PJ, Bednarkiewicz A (2021). Standardization of methodology of light-to-heat conversion efficiency determination for colloidal nanoheaters. ACS Appl Mater Interfaces.

[26] Liu X, Li B, Fu F, Xu K, Zou R, Wang Q (2014). Facile synthesis of biocompatible cysteine-coated CuS nanoparticles with high photothermal conversion efficiency for cancer therapy. Dalton Trans.

[27] Kuniyil Ajith Singh M, Steenbergen W (2015). Photoacoustic-guided focused ultrasound (PAFUSion) for identifying reflection artifacts in photoacoustic imaging. Photoacoustics..

[28] Nguyen HNY, Hussain A, Steenbergen W (2018). Reflection artifact identification in photoacoustic imaging using multi-wavelength excitation. Biomed Opt Express.

[29] Nguyen HNY, Steenbergen W (2019). Reducing artifacts in photoacoustic imaging by using multi-wavelength excitation and transducer displacement. Biomed Opt Express.

[30] Saha A, Ellison D, Thomas GJ, Vallath S, Mather SJ, Hart IR (2010). High-resolution in vivo imaging of breast cancer by targeting the pro-invasive integrin alphavbeta6. J Pathol.

[31] Gering DT, Nabavi A, Kikinis R, Hata N, O'Donnell LJ, Grimson WE (2001). An integrated visualization system for surgical planning and guidance using image fusion and an open MR. J Magn Reson Imaging.

[32] Desgrosellier JS, Cheresh DA (2010). Integrins in cancer: biological implications and therapeutic opportunities. Nat Rev Cancer.

[33] Schumacher S, Dedden D, Nunez RV, Matoba K, Takagi J, Biertümpfel C (2021). Structural insights into integrin α(5)β(1) opening by fibronectin ligand. Sci Adv.

[34] Barthelmes J, Dünnhaupt S, Unterhofer S, Perera G, Schlocker W, Bernkop-Schnürch A (2013). Thiolated particles as effective intravesical drug delivery systems for treatment of bladder-related diseases. Nanomedicine (Lond).

[35] Sogias IA, Williams AC, Khutoryanskiy VV (2008). Why is chitosan mucoadhesive?. Biomacromol.

[36] Kolawole OM, Lau WM, Mostafid H, Khutoryanskiy VV (2017). Advances in intravesical drug delivery systems to treat bladder cancer. Int J Pharm.

[37] Ren J, Xu S, Guo D, Zhang J, Liu S (2014). Increased expression of α5β1-integrin is a prognostic marker for patients with gastric cancer. Clin Transl Oncol.

[38] Xu Y, Li Y, Pan J, Kang X, Zhang X, Feng X (2020). EM2D9, A monoclonal antibody against integrin α5β1, has potent antitumor activity on endometrial cancer in vitro and in vivo. Cancer Lett.

[39] Yao H, Veine DM, Livant DL (2016). Therapeutic inhibition of breast cancer bone metastasis progression and lung colonization: breaking the vicious cycle by targeting α5β1 integrin. Breast Cancer Res Treat.

