# Supplementary material for: A simple and robust nanosystem for photoacoustic imaging of bladder cancer based on α5β1-targeted gold nanorods
Source: J Nanobiotechnology. 2023 Aug 27;21:301. doi: 10.1186/s12951-023-02028-5 (PMC10463347; doi:10.1186/s12951-023-02028-5)
Supplement: Supplementary file 1 — Additional file 1: Table S1. Biochemical characterization of peptide Iso4 by Ellman’s assay, electrospray ionization mass spectrometry (ESI–MS) analysis, RP-HPLC, nuclear magnetic resonance (NMR) spectroscopy and integrin binding. Table S2. Expression of cell surface integrins on murine MB49-Luc bladder carcinoma cells as determined by FACS analysis. Figure S1. Zoom into the 1H-1D NMR spectrum of peptide Iso4 centered on the Hα resonances of phg and Phg. NMR spectroscopy shows that peptide Iso4 consists of two isomers corresponding to a cyclic head-to-tail peptide with D-phenylglycine (phg) and L-phenylglycine (Phg), the latter accounting for about 30%. The HαD and HαL signals of phenylglycine (each giving rise to a doublet) are indicated. Figure S2. Binding of Iso4-HRP conjugate to microtiter plates coated with or without α5β1, αvβ3 and αvβ5. A Schematic representation of head-to-tail cyclized Iso4 and control peptides (Iso3 and ARA, positive and negative control, respectively). B Binding of peptide-HRP conjugates to microtiter plates coated with α5β1, αvβ3 and αvβ5. Peptide-HRP conjugates were prepared by coupling peptides to maleimide-activated horseradish peroxidase (HRP), as described in Ref. [15]. The binding assay was performed as described previously [15] using the indicated amount of integrins for microtiter plate coating. Mean ± SE, n = 2 wells. The Iso3-HRP and ARA-HRP were used as positive and negative controls, respectively, to assess integrin functionality (Iso3-HRP) and binding specificity (ARA-HRP). The binding curves of Iso4-HRP and ARA-HRP α5β1 are reprinted with the permission of Ref. [15]. Figure S3. Expression of α5-, β1-, β3-, β5-, αv-integrin subunits, and αvβ6 integrin on murine bladder MB49-Luc carcinoma cells. Integrin expression by MB49-Luc cells was analyzed by FACS using the indicated anti-integrin antibodies (5 µg/ml), and appropriate species-specific Alexa Fluor 488-labeled secondary antibodies (5 µg/ml). Binding of an isotype control [file 12951_2023_2028_MOESM1_ESM.docx]

**Supplemental Material**

**A simple and robust nanosystem for photoacoustic imaging of bladder cancer based on α5β1-targeted gold nanorods**

**Supplemental Table S1: Biochemical characterization of peptide Iso4 by Ellman’s assay, electrospray ionization mass spectrometry (ESI-MS) analysis, RP-HPLC, nuclear magnetic resonance (NMR) spectroscopy and integrin binding.**

| **Peptide**  **code** | **Peptide concentration**  ***by***  ***Ellman’s assay*** | **Molecular mass**  ***by***  ***ESI-MS*** | **Peptide**  **purity**  ***by***  ***RP-HPLC*** | **D-phg**  **content**  ***by***  ***NMR*** |  | **Affinity for integrin**  ***by***  ***competitive binding assay*** ^c^  (*Ki, nM*) | | | | |
| --- | --- | --- | --- | --- | --- | --- | --- | --- | --- | --- |
|  | (*mg/ml*) | (*Da, MH^+^)* | (*%*) | (%) |  | α5β1 | αvβ6 | αvβ8 | αvβ5 | αvβ3 |
| Iso4 | 8.380 ± 0.156 *^a^* | 622.24 ^b^ | > 95 | ~ 70 |  | 15 | 46 | 51 | 1121 | 1493 |

1. The lyophilized peptide was resuspended at 10 mg/ml, based on the gross weight. Mean±SD of two independent quantification.
2. Expected (Da, MH^+^): 622.32.
3. *Ki*, inhibitory constant values. Reprinted with the permission of Ref. 18.

**Supplemental Table S2: Expression of cell surface integrins on murine MB49-Luc bladder carcinoma cells as determined by FACS analysis.**

| **Anti-integrin mAb** | | | | |  | **Binding of anti-integrin mAb to MB49-Luc cells** | |
| --- | --- | --- | --- | --- | --- | --- | --- |
| *Antigen* | *Clone* | *Host* ^a^ | *Isotype* | *Species*  *reactivity* ^b^ |  |  | |
|  |  |  |  |  |  | *n ^c^* | *Fold increase ^d^* |
| α5 subunit | HMα5-1 | *Ha* | IgG | *M, R* |  | 2 | 3.79 ± 0.18 |
| β1 subunit | HMβ1-1 | *Ha* | IgG | *M, R* |  | 2 | 8.02 ± 1.04 |
| β3 subunit | HMβ3-1 | *Ha* | IgG | *M, R* |  | 2 | 1.02 ± 0.01 |
| β5 subunit | KN52 | *M* | IgG_1_ | *Hu, M, R, Ha* |  | 2 | 1.04 ± 0.28 |
| αv subunit | RMV-7 | *R* | IgG_1_ | *M* |  | 1 | 3.48 |
| αvβ6 | 10D5 | *M* | IgG_2a_ | *Hu, M, R* |  | 2 | 1.61 ± 0.78 |

*a) R*, rat; *M*, mouse; *Ha*, Armenian hamster; *Hu*, human.

b) According to the technical data sheet or published data.

c) *n*, number of independent experiments, each in duplicate.

d) *Fold increase* corresponds to the ratio of the mean fluorescence intensity of a given anti-integrin antibody over the mean fluorescence intensity of an isotype control matched antibody (mean ± SEM).

e) *Na*, not applicable.

**Supplemental Figures**


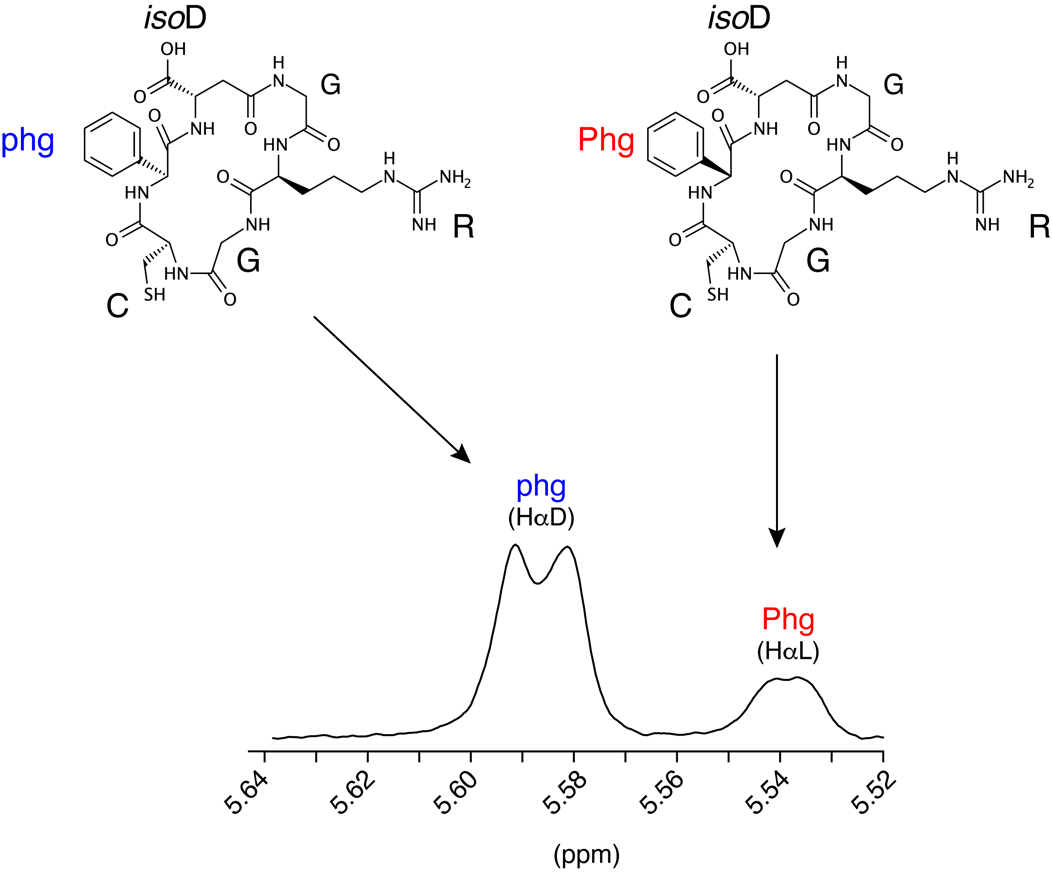


**Figure S1. Zoom into the ^1^H-1D NMR spectrum of peptide Iso4 centered on the Hα resonances of phg and Phg**

NMR spectroscopy shows that peptide Iso4 consists of two isomers corresponding to a cyclic head-to-tail peptide with D-phenylglycine (phg) and L-phenylglycine (Phg), the latter accounting for about 30%. The HαD and HαL signals of phenylglycine (each giving rise to a doublet) are indicated.

**
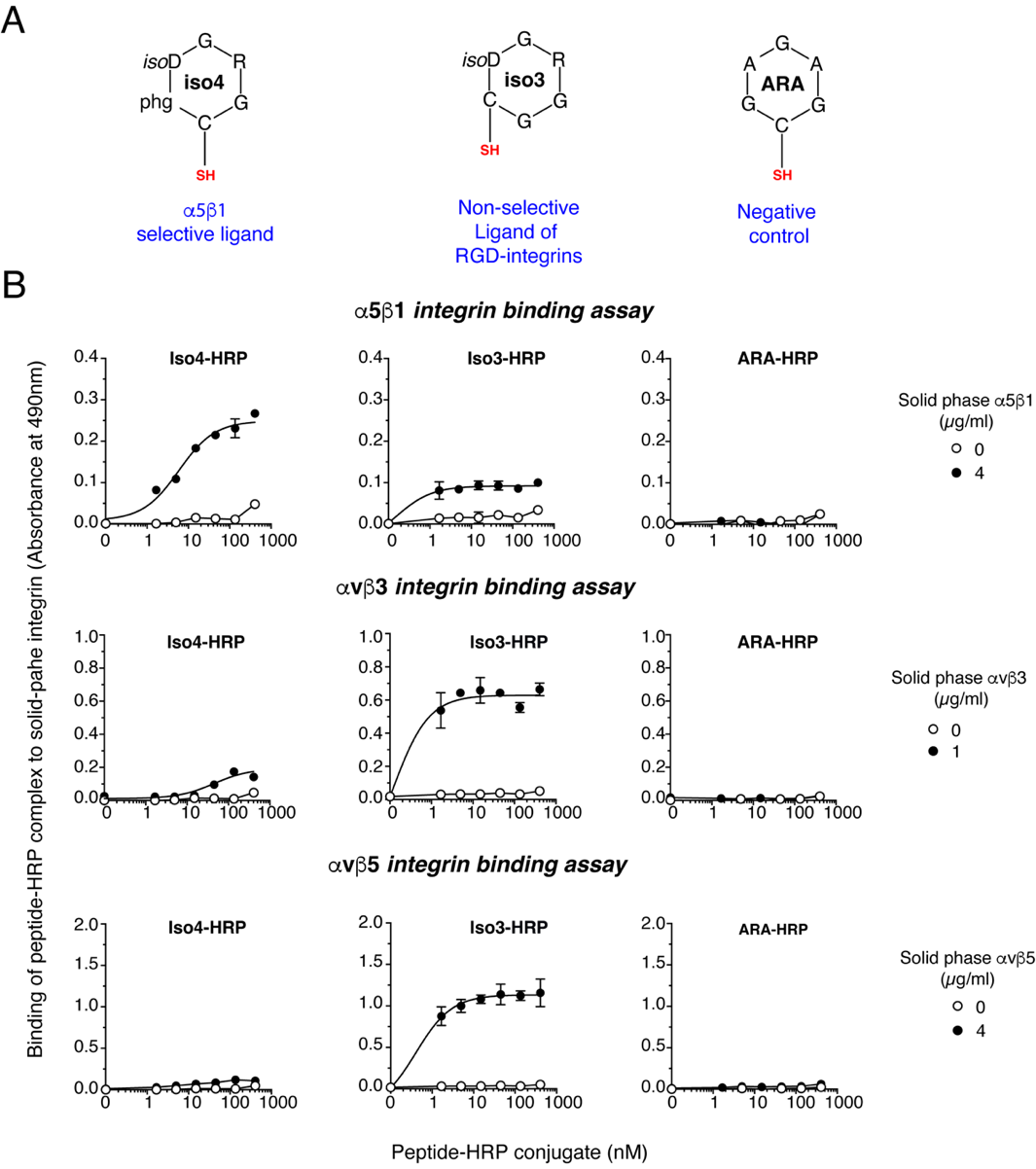
**

**Figure S2: Binding of Iso4-HRP conjugate to microtiter plates coated with or without α5β1, αvβ3 and αvβ5.**

**A**) Schematic representation of head-to-tail cyclized Iso4 and control peptides (Iso3 and ARA, positive and negative control, respectively).

**B**) Binding of peptide-HRP conjugates to microtiter plates coated with α5β1, αvβ3 and αvβ5. Peptide-HRP conjugates were prepared by coupling peptides to maleimide-activated horseradish peroxidase (HRP), as described in Ref. 15. The binding assay was performed as described previously (Ref. 15) using the indicated amount of integrins for microtiter plate coating. Mean±SE, n=2 wells. The Iso3-HRP and ARA-HRP were used as positive and negative controls, respectively, to assess integrin functionality (Iso3-HRP) and binding specificity (ARA-HRP). The binding curves of Iso4-HRP and ARA-HRP α5β1 are reprinted with the permission of Ref. 15.


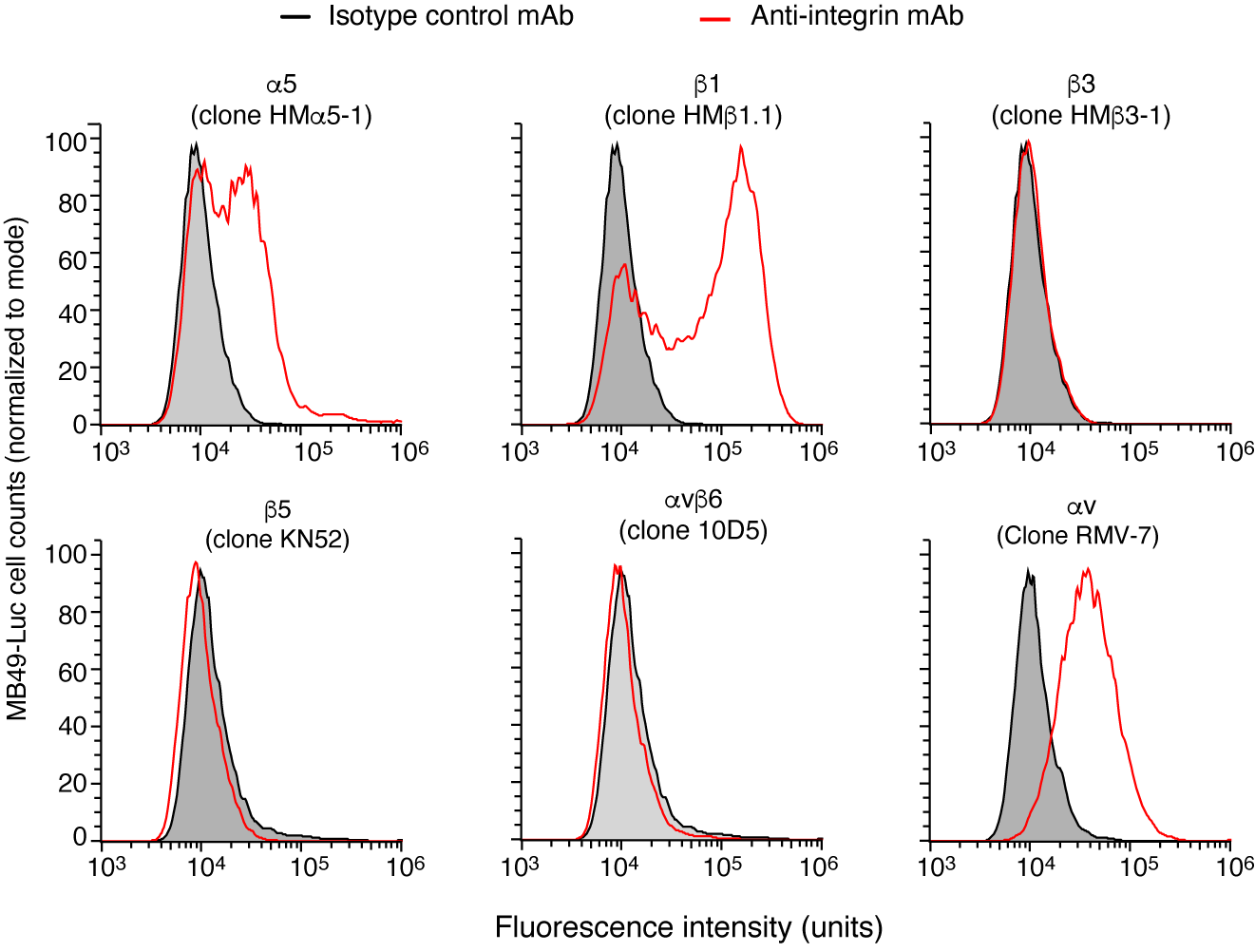


**Figure S3**. **Expression of α5-, β1-, β3-, β5-, αv-integrin subunits, and αvβ6 integrin on murine bladder MB49-Luc carcinoma cells.**

Integrin expression by MB49-Luc cells was analyzed by FACS using the indicated anti-integrin antibodies (5 µg/ml), and appropriate species-specific Alexa Fluor 488-labeled secondary antibodies (5 µg/ml). Binding of an isotype control antibody is also shown (see Supplemental **Table S2** for antibody description).

**­­
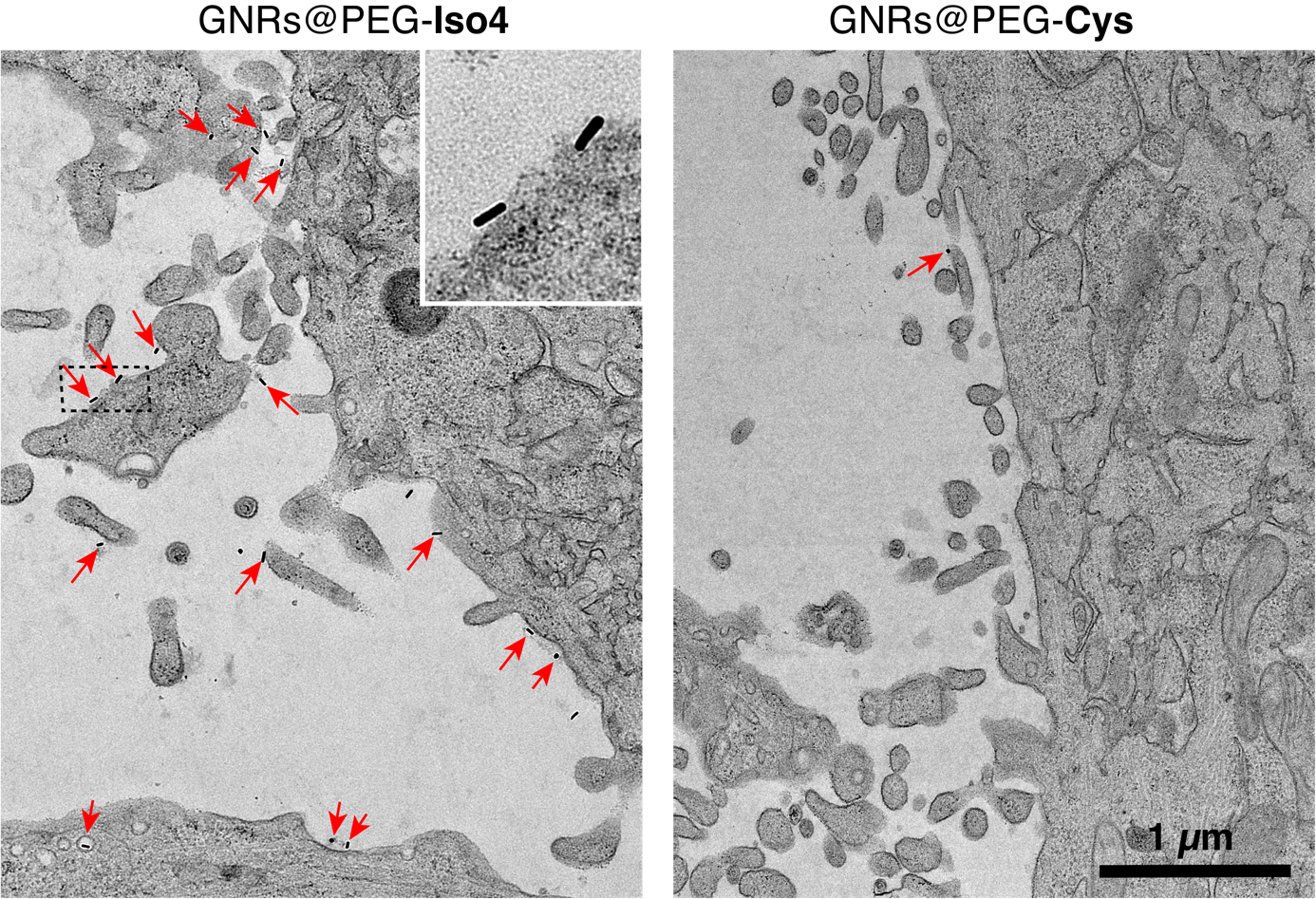
**

**Figure S4. TEM analysis of MB49-Luc cells incubated with GNRs@PEG-Iso4 or GNRs@PEG-Cys.**

MB49-Luc cells, cultured in a 12-well plate (cell confluency >90%), were washed twice with 0.9% sodium chloride and then incubated for 5 min with 25 mM Hepes buffer, pH 7.4, containing 150 mM sodium chloride, 1 mM magnesium chloride, 1 mM manganese chloride, 1% w/v BSA. The cells were then incubated for 2 h at 37 °C, 5% CO_2_, with GNRs@PEG-Iso4 or GNRs@PEG-Cys (1x10^11^ NPs/ml, 500 μl well). The cells were then washed with the same buffer (3 times, 5 min each), fixed, and analyzed by TEM as described in supplemental ref. (1).


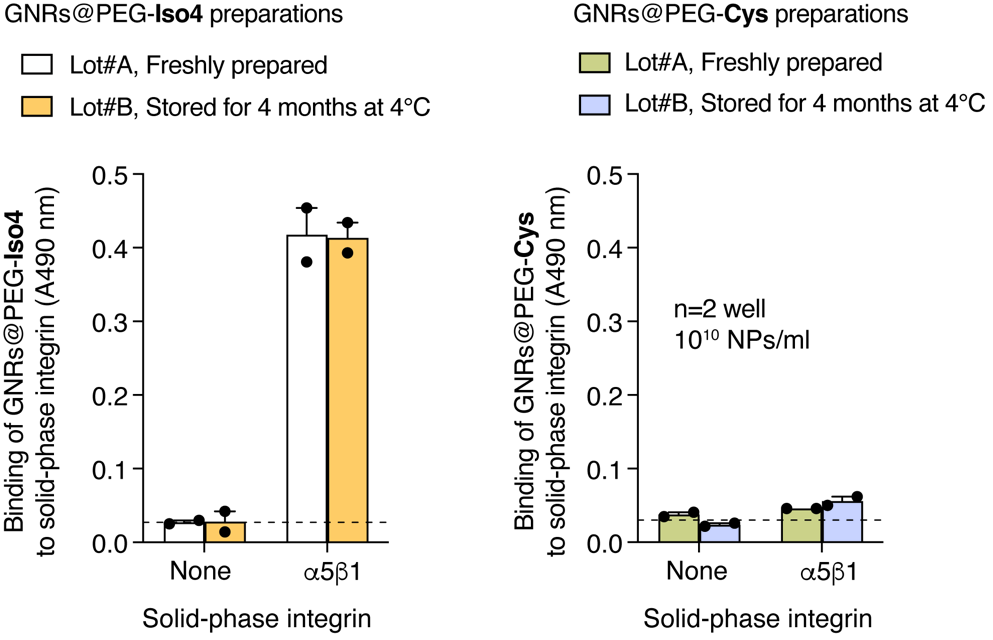


**Figure S5. Characterization of GNRs@PEG-Iso4 stability by α5β1-integrin binding assay.**

Binding of GNRs@PEG-Iso4 (different lots*,* called *#A* and *#B*, corresponding to freshly prepared NPs or after 4 months of storage at 4°C, respectively), to microtiter plated coated with or without α5β1. NPs binding was detected as described in *Methods*. GNRs@PEG-Cys was also included as a negative control. Note that the binding of GNRs@PEG-Iso4 Lot#B is similar to that of Lot#A, suggesting that no chemical detachment or degradation of the various compounds composing the NPs occurred during the storage for 4 months at 4°C.


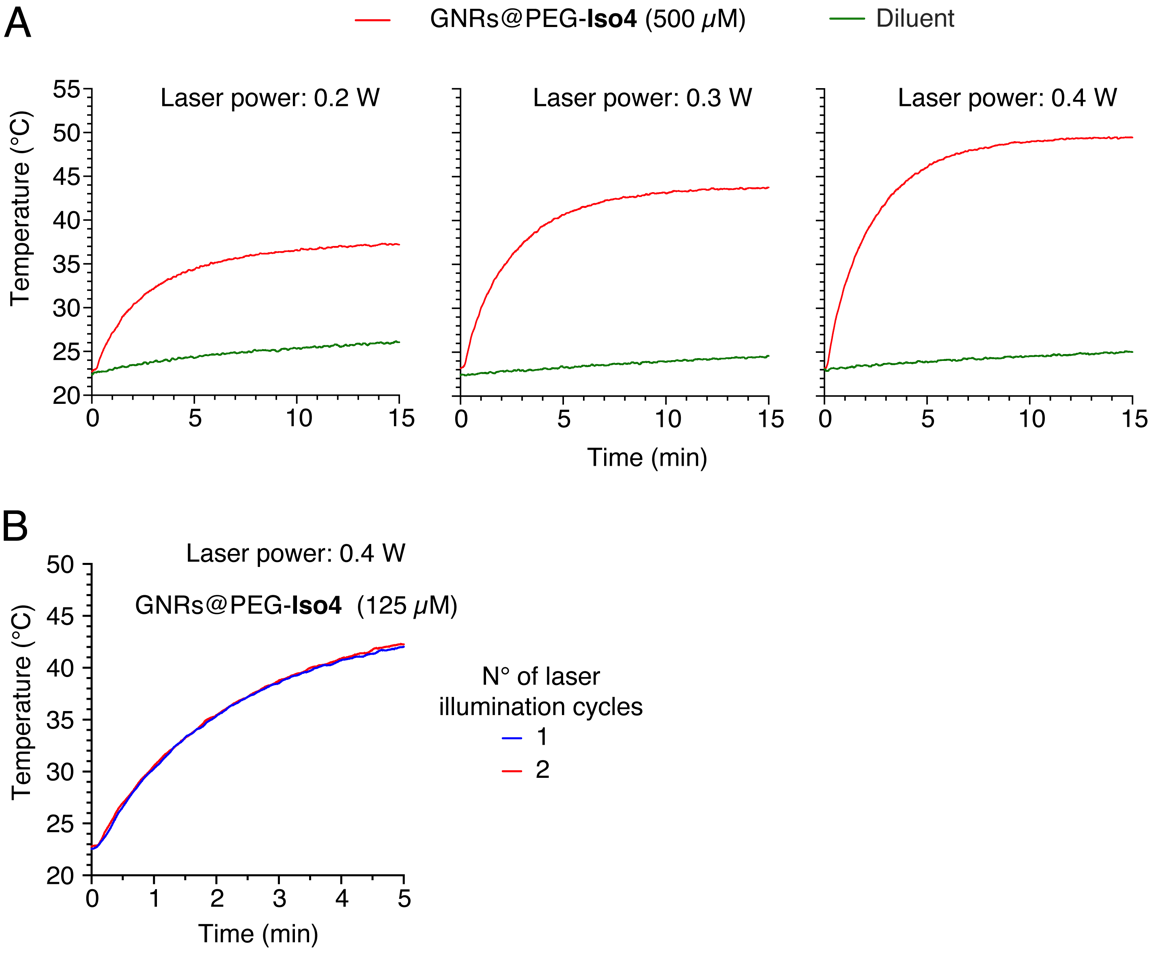


**Figure S6. Photothermal properties of GNRs@PEG-Iso4.**

The capability of GNRs@PEG-Iso4 to release heat after illumination (0-15 min) was tested using a custom-made system consisting of: *1*) a continuous-wave NIR laser line at 808 nm (LDC220C, TED200C, Thorlabs, laser power, 0.2, 0.3 and 0.4 W), *2)* a multimodal optical fiber (1 mm Ø, Thorlabs), *3*) a power meter sensor (Thorlabs, cat. s405c); *4*) a cuvette holder (Thorlabs), *5*) a polystyrene cuvette (Kartell, cat. 01938-00) containing 500 µl of sample, (0-500 µM of Au, final concentration) and *6*) a NIR camera (HEIMANN Sensor, cat. HTPA80x64dR2L3.9/0.8HiA).

**A**) Heating curve of GNRs@PEG-Iso4 (500 µM Au) dispersed in 0.05% HSA (*Diluent*) and irradiated with an 808 nm laser at the indicated power densities. *Right panel inset*, the image of the cuvette containing GNRs@PEG-Iso4 after 15 min of laser illumination at 0.4 W is shown.

**B**) Thermal stability of GNRs@PEG-Iso4 (125 µM Au) over 2 cycles of a laser on/off experiment at the indicated laser power. The 2^nd^ cycle of a laser illumination was performed after 24 h from the 1^st^ cycle.


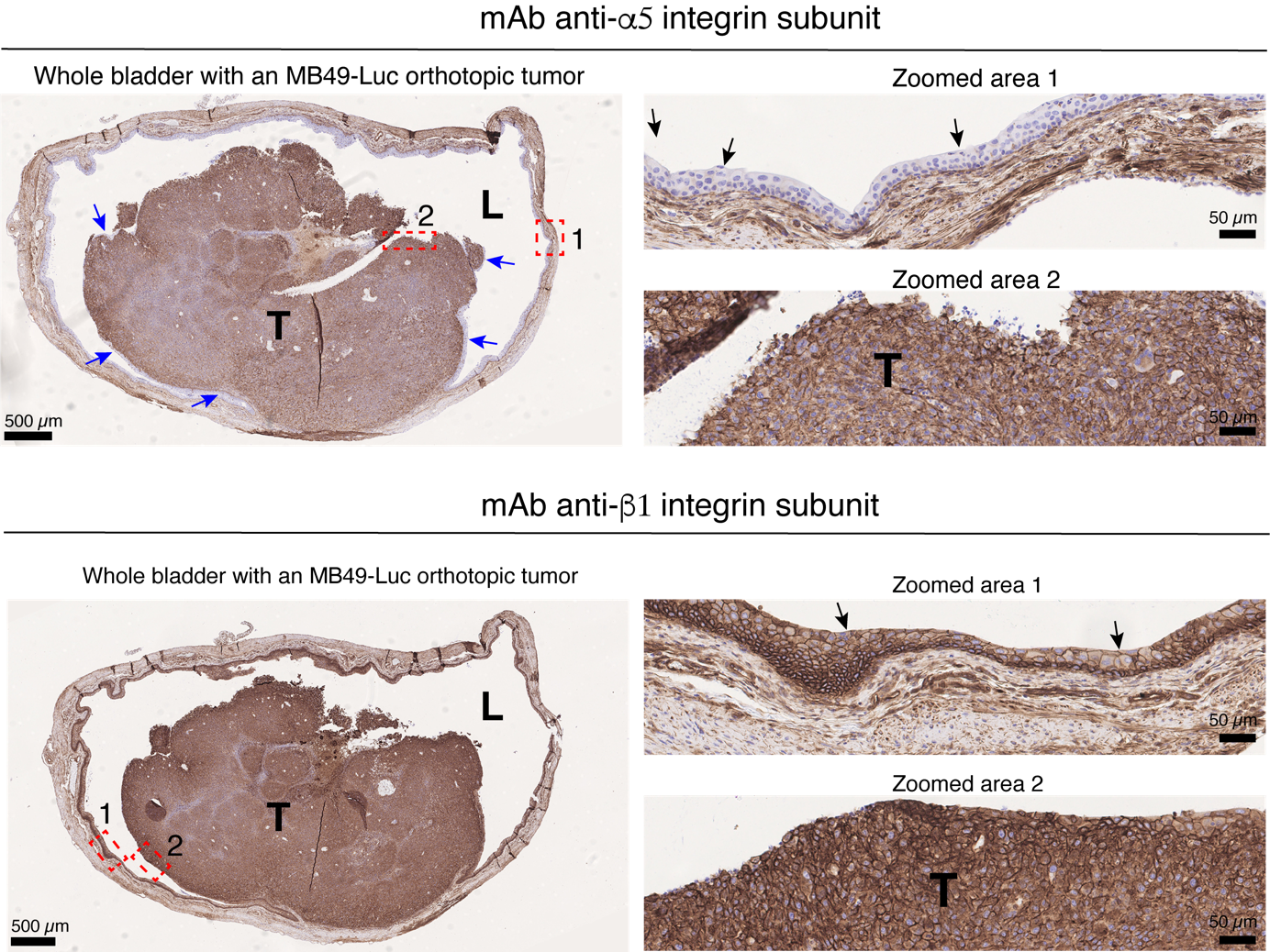


**Figure S7. Expression of α5- and β1-integrin subunit in the MB49-Luc bladder cancer model.**

Representative immunohistochemistry photomicrographs of the expression of α5- and β1-integrin subunit in different areas of the bladder of a tumor-bearing mice, 15 days after intravesical instillation of MB49-Luc cells

*T*, MB49-Luc tumor; *L*, lumen of the urinary bladder; *Red* *dashed rectangle*: zoomed areas. *Zoomed area 1*: healthy bladder, *black* *arrows* indicate the urothelial cells. *Zoomed area 2*: Luminal side of MB49-Luc tumor. Note that this big tumor is minimally covered by layers of α5-negative urothelial cells (*blue* *arrows)*. Immunostaining was performed as described previously (15). Adapted and reprinted with the permission of Ref (15).


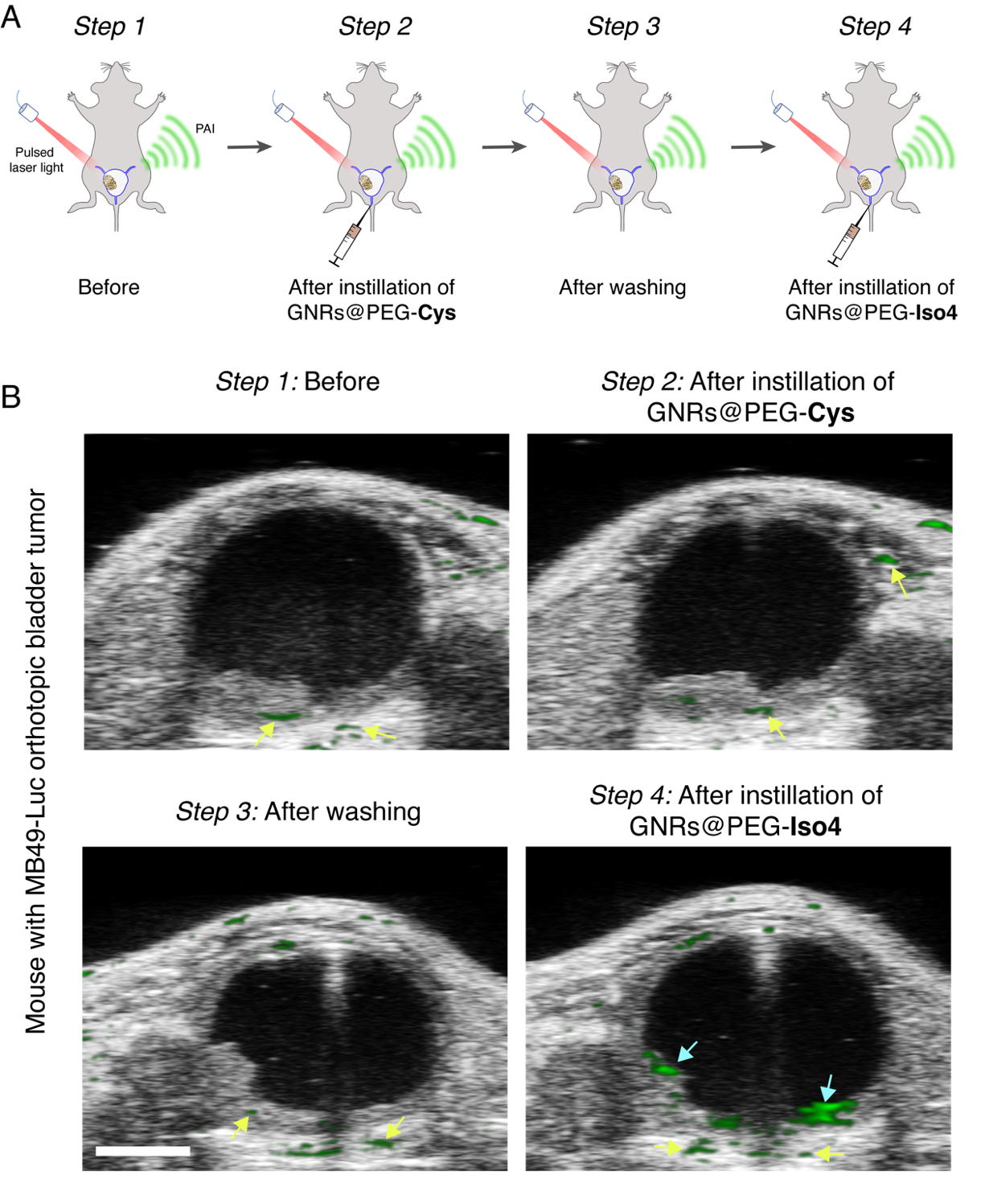


**Figure S8. GNRs@PEG-Cys does not bind to orthotopic MB49-Luc tumor lesions.**

**A**) Schematic representation of the experimental procedure. A mouse with orthotopic MB49-Luc tumor lesions underwent PAUS imaging of the bladder before (*Step 1*) and after (*Step 2)* intravesical instillation of GNRs@PEG-Cys (26 nmol Au). After bladder washing, additional PAUS imaging of the bladder was then performed before (*Step 3*) and after (*Step 4*) instillation of GNRs@PEG-Iso4 (26 nmol Au).

**B**) Representative PAUS images (axial 2D) of the bladder after step 1, 2, 3, and 4. *Grayscale*, co-registered US signal; *Green*, PA signal; *Cyan arrows*, specific PA signal (generated by GNRs@PEG-Iso4). *Yellow arrows*, unspecific PA signal (independent from nanoparticles). *Bar*, 2 mm. Specific PA signal on tumor lesions was observed only after step 4, suggesting that peptide Iso4 is crucial for tumor recognition by nanoparticles.


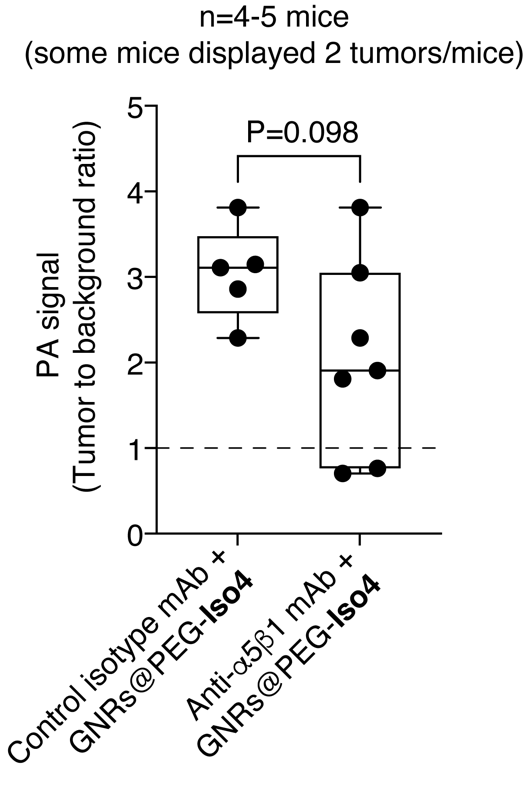


**Figure S9. Effect of a neutralizing anti-α5β1 mAb on the uptake of GNRs@PEG-Iso4 to MB49-Luc bladder tumors.**

Mice bearing orthotopic MB49-Luc tumors were intravescically administered with a control isotype mAb or a neutralizing anti-α5β1 mAb (clone: RTK2758 and 5H10-27(MFR5), respectively, 20 µg/mouse). After 15 min, the bladders were emptied and subsequently filled with GNRs@PEG-Iso4 (26 nmol Au in 100 µl, ~ 1x10^11^ NPs). After 15 min, the bladders were washed, and PA and US imaged. PA signals associated with whole tumors or adjacent healthy tissues (*background*) were quantified using VevoLab 5.6.1 software. Box-plots with median, interquartile and 5-95 percentile, in which dots represent well-established tumors. N=4-5 mice, with one or two tumors per bladder. *P*, by Mann–Whitney nonparametric test.

**Supplemental References**

1. Carsana EV, Lunghi G, Prioni S, Mauri L, Loberto N, Prinetti A, et al. Massive Accumulation of Sphingomyelin Affects the Lysosomal and Mitochondria Compartments and Promotes Apoptosis in Niemann-Pick Disease Type A. J Mol Neurosci. 2022;72(7):1482-99.
